# Unprecedented Burning in Tropical Peatlands During the 20th Century Compared to the Previous Two Millennia

**DOI:** 10.1111/gcb.70717

**Published:** 2026-03-17

**Authors:** Yuwan Wang, Ted R. Feldpausch, Graeme T. Swindles, Patrick Moss, Hamish A. McGowan, Thomas G. Sim, Paul J. Morris, Adam Benfield, Colin Courtney‐Mustaphi, David Wahl, Encarni Montoya, Esther Githumbi, Eurídice N. Honorio Coronado, Femke Augustijns, Gert Verstraeten, Jess O' Donnell (Roe), John Tibby, Juan C. Benavides, K. Anggi Hapsari, Karsten Schittek, Khairun Nisha Mohamed Ramdzan, Kunshan Bao, Lydia E. S. Cole, Lysanna Anderson, Mariusz Gałka, Orijemie Emuobosa Akpo, Paul Strobel, Prabhakaran Ramya Bala, René Dommain, Rob Marchant, Raman Sukumar, Sakonvan Chawchai, Sarath Pullyottum Kavil, Scott Mooney, Thomas J. Kelly, Yang Gao, Apostolos Voulgarakis, Arnoud Boom, Chantelle Burton, Juan Carlos Berrio, Kelly Ribeiro, Liana O. Anderson, Mark Hardiman, Molly Spater, Susan E. Page, Angela V. Gallego‐Sala

**Affiliations:** ^1^ Department of Geography University of Exeter Exeter UK; ^2^ School of the Environment University of Queensland Brisbane Australia; ^3^ State Key Laboratory of Lithospheric and Environmental Coevolution, Institute of Geology and Geophysics, Chinese Academy of Sciences Beijing China; ^4^ Geography, School of Natural and Built Environment Queen's University Belfast Belfast UK; ^5^ Ottawa‐Carleton Geoscience Centre and Department of Earth Sciences Carleton University Ottawa Ontario Canada; ^6^ School of Earth & Atmospheric Sciences Queensland University of Technology Brisbane Australia; ^7^ Weather and Climate Science Research Alliance The University of Queensland Brisbane Australia; ^8^ Forest Research, Northern Research Station Roslin Midlothian UK; ^9^ School of Geography University of Leeds Leeds UK; ^10^ Department of Earth and Environment Franklin and Marshall College Lancaster Pennsylvania USA; ^11^ Department of Geological and Environmental Scienc Appalachian State University Boone North Carolina USA; ^12^ Geoecology, Department of Environmental Sciences University of Basel Basel Switzerland; ^13^ Department of Geography and Environment, York Institute for Tropical Ecosystems University of York York UK; ^14^ Center for Water Infrastructure and Sustainable Energy (WISE) Futures, Nelson Mandela African Institution of Science and Technology Arusha Tanzania; ^15^ Knowledge Core LLC Basel Basel‐Stadt Switzerland; ^16^ U.S. Geological Survey, Geology, Minerals, Energy, and Geophysics Science Center Menlo Park California USA; ^17^ Geosciences Barcelona, CSIC, c/LLuis Solé i Sabaris s/n Barcelona Spain; ^18^ Institute of Soil Science and Site Ecology TU Dresden Tharandt Germany; ^19^ Royal Botanic Gardens, Kew London UK; ^20^ Division of Geography and Tourism, Department of Earth and Environmental Sciences KU Leuven Leuven Belgium; ^21^ School of Biological, Earth & Environmental Sciences The University of New South Wales Sydney New South Wales Australia; ^22^ Geography, Environment and Population University of Adelaide Adelaide South Australia Australia; ^23^ Department of Ecology and Territory Pontificia Universidad Javeriana Bogotá Colombia; ^24^ Albrecht‐von‐Haller Institute University of Goettingen Goettingen Germany; ^25^ Institute of Geography Education University of Cologne Köln Germany; ^26^ Earth Observatory of Singapore Nanyang Technological University Singapore Singapore; ^27^ School of Geographic Science South China Normal University Guangzhou China; ^28^ School of Geography & Sustainable Development University of St Andrews Fife UK; ^29^ Faculty of Biology and Environmental Protection, Department of Biogeography, Paleoecology and Nature Conservation University of Lodz Łodz Poland; ^30^ Department of Archaeology and Anthropology University of Ibadan Ibadan Nigeria; ^31^ Department of Physical Geography Friedrich Schiller University Jena Jena Germany; ^32^ National Institute of Advanced Studies, Indian Institute of Science Campus Bengaluru India; ^33^ University of Potsdam Institute of Geosciences Potsdam‐Golm Germany; ^34^ Human Origins Program, National Museum of Natural History Smithsonian Institution Washington DC USA; ^35^ Faculty of Environment and Resource Studies Mahidol University Nakhon Pathom Thailand; ^36^ Centre for Ecological Sciences Indian Institute of Science Bangalore India; ^37^ Department of Geology, Faculty of Science Chulalongkorn University Bangkok Thailand; ^38^ Department of Geological Sciences Stockholm University Stockholm Sweden; ^39^ School of Geography Queen Mary University of London London UK; ^40^ School of Karst, Guizhou Normal University/State Engineering Technology Institute for Karst Desertification Control Guiyang China; ^41^ Leverhulme Centre for Wildfires, Environment and Society, Department of Physics Imperial College London London UK; ^42^ School of Chemical and Environmental Engineering Technical University of Crete Chania Greece; ^43^ School of Geography, Geology and the Environment University of Leicester Leicester UK; ^44^ Met Office Hadley Centre, Met Office Exeter UK; ^45^ National Institute for Space Research (INPE) São Paulo Brazil; ^46^ National Center for Monitoring and Early Warning of Natural Disasters (CEMADEN) São José dos Campos Brazil; ^47^ School of the Environment and Life Sciences University of Portsmouth Portsmouth UK; ^48^ Department of Geography & Planning University of Liverpool Liverpool UK

**Keywords:** charcoal, contemporary fire, last two millennia, palaeoenvironment, paleofire, tropical peatland

## Abstract

Tropical peatland wildfire incidence has risen in recent decades, driven by drainage for land use and intensified by severe droughts with global climate change. These disturbances have altered vegetation structure, disrupted ecosystem functioning, and increased carbon emissions, particularly in Southeast Asia. However, the long‐term history and characteristics of wildfires in tropical peatlands remain largely unknown. Here, we compiled fifty‐eight macro‐charcoal records from peatlands across the tropics, ranging from lowland forested to montane peatlands, to assess millennia‐scale changes and controlling factors of tropical peatland burning. We divided the datasets into four main sub‐regions: Neotropical, Afrotropical, Indomalayan and Australasian ecoregions to explore regional variability. Tropical peatlands had high burning levels between 0 and 850 ce, followed by a relatively low and stable period until a marked increase during the 20th century. The general trend in tropical peatland burning follows changes in global temperature, and climate variables that control the length and severity of drought events have a notable influence on peat burning before 1900 ce. During the 20th century, regional differences were observed, with declining fire trends in the Neotropical and Afrotropical regions and increasing fire trends in the Indomalayan and Australasian regions. This difference is likely attributable to human activities, and such intervention is also evident in palm swamps and hardwood swamps under similar wet, weakly seasonal climates. With the increase in anthropogenic pressures on peatlands and greater climate variability, future wildfires in peatlands are likely to become more frequent and widespread across all tropical ecoregions. Conservation and sustainable land‐use practices could be used to mitigate and control peatland burning and protect these carbon‐rich sinks.

## Graphical abstract

This study reveals unprecedented burning in tropical peatlands during the 20th century, mainly expressed in the Indomalayan and Australasian regions, emphasising regional variability in peatland burning and its links to modern human activities. Before this period, tropical peatland burning largely followed changes in global temperature and was influenced by climate variables that control the duration and severity of droughts over the past two millennia. These findings highlight the importance of conservation and sustainable land‐use practices as key strategies for mitigating future peatland wildfires.

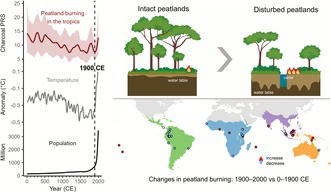

## 1 Introduction

Peatlands are biodiverse ecosystems that play a critical role in regulating the global carbon cycle over millennia (Page et al. 2011), with the most extensive and better‐studied peatlands located at high latitudes (Gorham 1991; Yu et al. 2010). Although the understanding of tropical peatlands has advanced in recent decades, including newly described peatlands in the Peruvian Amazon (Householder et al. 2012; Lähteenoja et al. 2012; Draper et al. 2014) and the Congo Basin (Dargie et al. 2017; Crezee et al. 2022), research on these ecosystems remains scarce (Joosten 2016). Tropical peatlands have received considerably more attention in recent years due to very large carbon losses under intensified human activities from deforestation, drainage, and land conversion to industrial oil palm, pulp plantations and other forms of agriculture (Page et al. 2022). These activities have been compounded by the influence of climate variability and extremes (e.g., drought and fires in El Niño years), as experienced in Southeast Asian peatlands (Page et al. 2002; Deshmukh et al. 2021). The threat from anthropogenic activities is likely to grow due to increased commercial and infrastructural demands (Roucoux et al. 2017; Dargie et al. 2019). This, combined with predicted future climate warming and changes to the hydrological cycle (Li et al. 2007; Wang et al. 2018; Tangang et al. 2020), could potentially alter intact peatlands and accelerate the deterioration of those already disturbed in the tropical region. Decreased return fire periods and increased fire intensities resulting from changes in climate and anthropogenic activities have been, and are expected to continue to jeopardise the stability of these carbon‐extensive ecosystems (Loisel et al. 2021).

Peatlands are naturally less susceptible to ignition than the wider landscape and they generally prevent the spread of fires because of their consistently high water tables, which also facilitate carbon accumulation. The flammability of the peat matrix varies with latitude and may correlate with its botanical composition (Crawford et al. 2024). However, disturbed peatlands are more prone to burning across all climate zones (Turetsky et al. 2015; Konecny et al. 2016). In the case of fires in disturbed peatlands, despite fuel load from aboveground biomass, dry peat can serve as extra fuel in smouldering combustion due to water drawdown (Usup et al. 2004; Rein 2013). Smouldering combustion of peat can persist for days to months or even longer (Rein 2016), causing large CO_2_ (Van Der Werf et al. 2017) and PM_2.5_ emissions with negative public health impacts (Kiely et al. 2020) and associated economic losses (Kiely et al. 2021). Peat accumulation can be hindered by the process of burning and material reworking (Clark and Patterson III 1997; Rius et al. 2011; Remy et al. 2018).

Charcoal is the product of incomplete combustion in vegetation and peat fires, and is widely used for identifying the occurrence of fire across different environments (Whitlock and Larsen 2001; Conedera et al. 2009). Different sizes of charcoal can provide valuable insights into fire patterns at different landscape scales with most macroscopic charcoal (typically defined as ≥ 100–250 μm in diameter (Vachula 2019)) depositing near the source of burning, and microscopic charcoal more likely to be transported further away (Clark 1988; Lynch et al. 2004). The synthesis of multiple charcoal records from a wider regional or even continental scale makes it possible to compile regional fire regimes for different biomes and disentangle fire drivers (Marlon et al. 2008; Power et al. 2008; Mooney et al. 2011). Previous studies based on diverse geological archives, which convey varying spatiotemporal scales and interpretations of fire history, have shown that charcoal preserved in peats is considered to reflect more localised burning (Whitlock and Anderson 2003; Conedera et al. 2009). There is a lack of studies exclusively focused on peatland fire regimes, and only one such compilation exists for mid‐ to high‐latitude peatlands (Sim et al. 2023).

The last 2000 years have been a period of intensifying human influence on global land cover with a general expansion of agricultural and grazing land and associated carbon emissions (Kaplan et al. 2011; Klein Goldewijk et al. 2017) while maintaining similar conditions to modern climate. Given that high‐resolution records are most available for this period, we chose this period to study the response of peatland burning to past climatic events and investigate how modern climate change is influencing peatland fire regimes.

Here, we present the first compilation of charcoal records from tropical peatlands over the last two millennia and address the following questions: (1) How have fire regimes changed over the last 2000 years in tropical peatlands and has peatland burning increased in the 20th century? (2) Does peatland burning exhibit different fire regimes compared to non‐peatland landscapes and does this vary by sub‐region? and (3) What are the controlling factors of tropical peatland burning during the last 2000 years?

## 2 Materials and Methods

### 2.1 Study Regions

We compiled macro‐charcoal records (> 100 μm) spanning the last two millennia (same as Sim et al. 2023) from peatlands in the tropical region (defined as the area between the subtropical latitudes of 30° N and 30° S). Though site Vankervelsvlei in South Africa is slightly outside the defined region, we included this site considering its potential to contribute valuable data in less‐studied areas. These macro‐charcoal records were assumed to reflect local fire activity (biomass burned and fire frequency (Marlon et al. 2009)), representing the burning of aboveground vegetation and/or peat soils, although we cannot rule out charcoal deposition from distant fires. Additionally, due to the absence of geochemical proxies in the compilation, we cannot exclude the effect of peat loss from severe in situ smouldering fires (Zaccone et al. 2014), though stratigraphic hiatuses in peat profiles may provide indirect clues to such peat loss (Magnan et al. 2020).

Forty out of 58 sites were collected under the HOLOPEATFIRE project (Sim et al. 2023), and the remaining sites were obtained from public charcoal datasets, including the Global Paleofire Database (formerly Global Charcoal Database) (Power et al. 2008), The Reading Palaeofire Database (Harrison et al. 2021), Neotoma Paleoecology Database (Williams et al. 2018), PANGAEA (Diepenbroek et al. 2002), and original publications to improve spatial coverage (Table S1). To enable the comparison across the tropics, sites were divided into four main geographical sub‐regions as described in Dinerstein et al. (2017): Neotropical, Afrotropical, Indomalayan and Australasian ecoregions with 16, 15, 16 and 8 sites, respectively. Three sites located in the Oceanian realm were included in the tropical compilation, but not used to generate a regional composite curve due to too few sites being available.

Peat is generally defined as organic soil with at least 30%–80% organic matter (Lourenco et al. 2023). In this study, the criterion for identifying peat layers was set at 30% organic matter (Joosten and Clarke 2002), or 15% carbon, a median value of 12%–18% organic carbon considering inorganic intrusion into the layers (Deckers and Nachtergaele 1998). When organic matter or carbon content was not explicit or available, we relied on the descriptions in the publication (*n* = 33). Any continuous minerogenic section was excluded from the peat profile to eliminate burning information from outside the peatland ecosystem via fluvial transport. Only when the peat profile was intercalated with very thin mineral layers, the entire peat profile was considered in the analysis.

### 2.2 Building Age‐Depth Models

New age‐depth models for each site were constructed based on age control data (i.e., top of the core, radiocarbon dates, ^210^Pb and ^137^Cs) using Bayesian methods. The *rbacon* (Blaauw and Christen 2011) package was used in most cases, whereas the *rplum* package was used instead when raw ^210^Pb data are available (Aquino‐López et al. 2018) (Figures S1–S8). All radiocarbon dates were calibrated based on SHCal20, IntCal20, or a 50:50 mixed curve of SHCal20 and IntCal20, depending on the location of the specific site (Hogg et al. 2020; Reimer et al. 2020). As a quality control criterion, we excluded any peat core that did not have at least two age control data points for the last 3000 years, which allows age interpolation for the past two millennia.

### 2.3 Charcoal Data Transformation and Synthesis for Peatland and Landscape Burning

Because the charcoal records differ in extraction techniques and quantification methods, the data required standardisation to enable comparison across sites. Proportional relative scaling (PRS) was selected to carry out all charcoal transformations, which is a method particularly suited for ecosystems with infrequent fires and/or when charcoal particles seem poorly recorded (McMichael et al. 2021). Firstly, charcoal concentrations (e.g., particles cm^−3^) were transformed to influxes (e.g., particles cm^−2^ year^−1^) by multiplying by the accumulation rate (cm year^−1^) obtained from age‐depth models. The influxes were then transformed using the following equation (McMichael et al. 2021):

$${\text{Char}}_{\mathrm{PRS}}=\left(\frac{C_{i}}{C_{\max}}\times 100\right)\times \frac{f}{N}$$

where Char_PRS_ is charcoal influx value after PRS transformation, *C*_i_ is charcoal influx value within a record, *C*_max_ is the maximum charcoal influx value within a record, *f* is the number of charcoal influx values (> 0) within a record, and *N* is the total number of charcoal influx values within a record.

Transformed charcoal influx values were then in the same range between 0 and 100 with higher values indicating more peatland burning. Composite curves were then constructed for sub‐regions after a two‐stage smoothing method that included: (a) individual records were binned every 25 years (median resolution across all sites), and (b) smoothed with a 200‐year window using locally weighted scatterplot smoother via the *paleofire* package (Blarquez et al. 2014). The confidence intervals were calculated by bootstrap resampling of the binned charcoal series and calculation of the mean for each bin 1000 times, and confidence limits for each target point were taken as the 5% and 95% percentiles.

**FIGURE 1:**
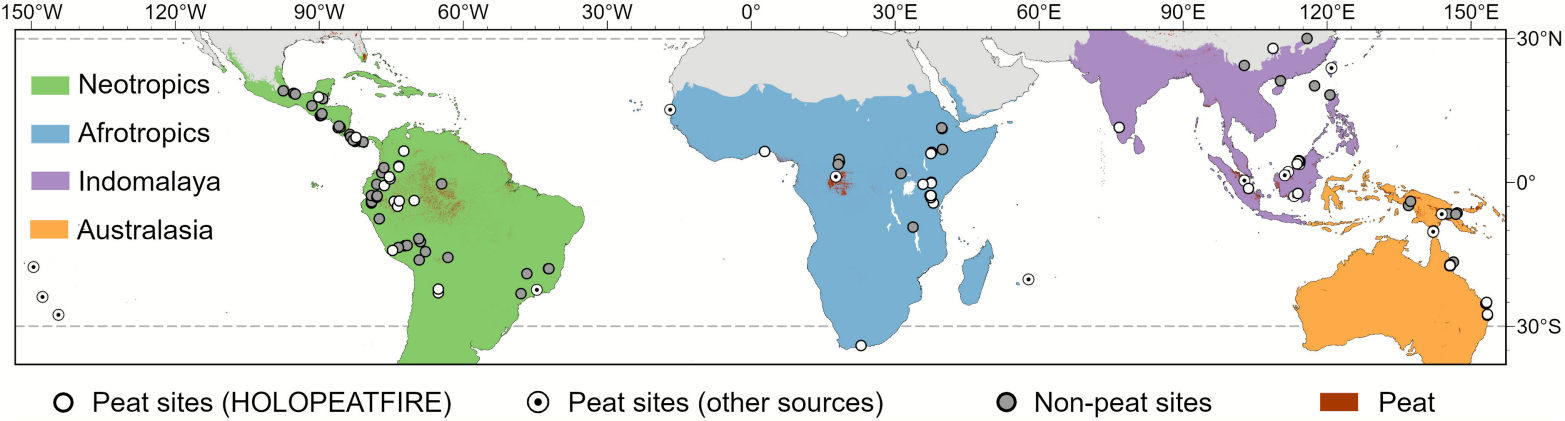
Map of study sites. White circles (with dots) indicate peat sites and grey circles represent sites involved in landscape burning from the Reading Palaeofire Database (Harrison et al. 2021), based on PEATMAP (red shaded area) (Xu et al. 2018) with different terrestrial realms (Olson and Dinerstein 2002). Map lines delineate study areas and do not necessarily depict accepted national boundaries.

To enable comparisons between peatland burning and wider non‐peatland landscape burning (hereafter referred to as landscape burning), we selected only charcoal records from non‐peatland landscapes from the Reading Palaeofire Database (Harrison et al. 2021) using an appropriate buffer around our peat sites. Three different buffers (200, 500 and 800 km) were tested in the Neotropical region since this region had the largest number of sites, and we found that landscape burning generated within the 800‐km buffer showed a similar signal to the composite curve using the other smaller ranges (*r* > 0.8 between 800 km and the other two buffers) (Figure S9). Thus, an 800‐km buffer was chosen for generating wider landscape burning to include more sites, this is especially important in data‐sparse regions. Ninety‐one sites were treated with the same charcoal transformation and composition procedure for each sub‐region and were assumed to provide information on wider landscape burning compared to peatland burning (Figure 1; Table S2) (Whitlock and Anderson 2003).

### 2.4 Recent Burning in the 20th Century Compared to the Last Two Millennia

**FIGURE 2:**
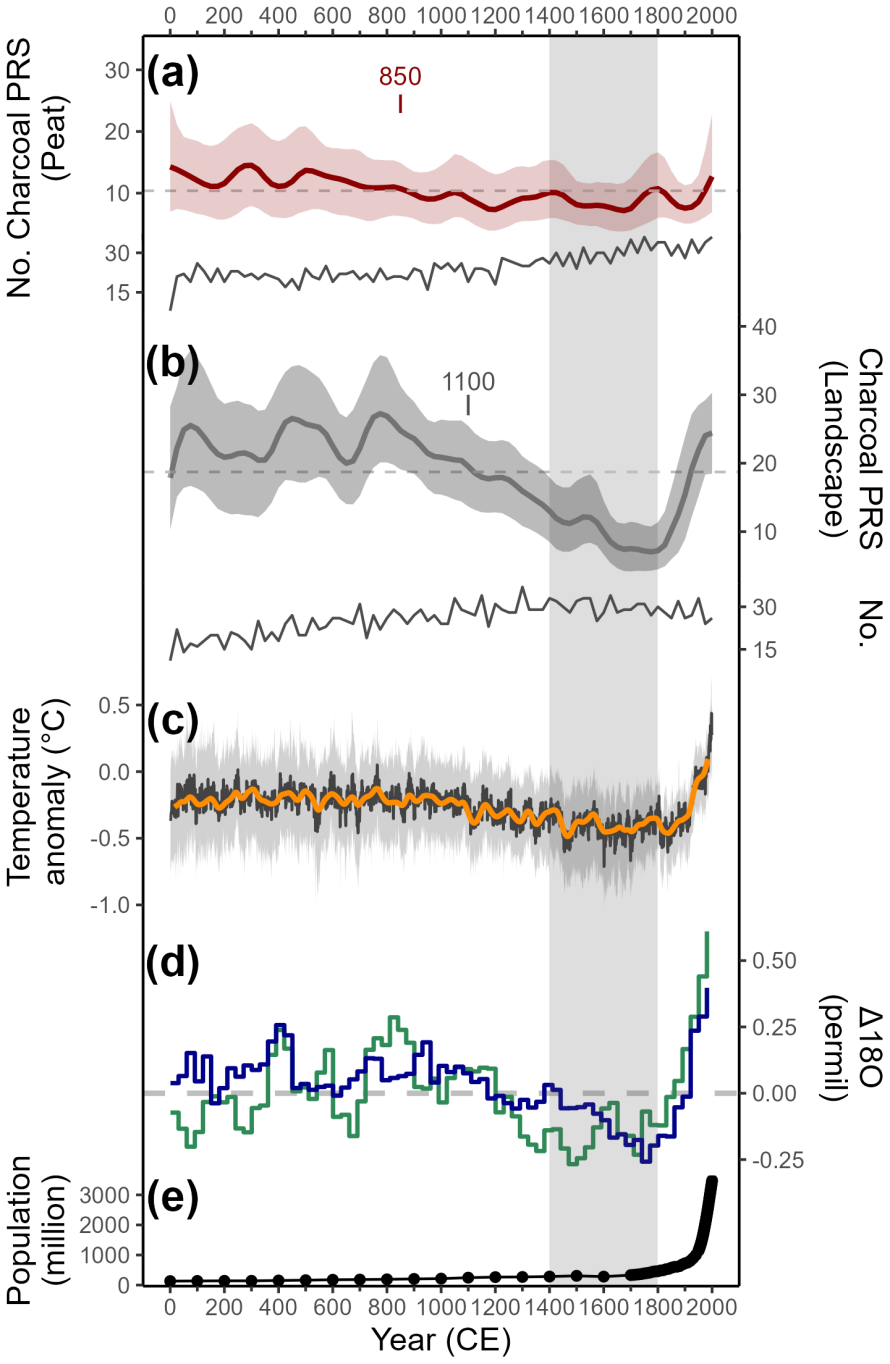
Reconstructions of biomass burning in the tropics with global temperature, hydroclimate and tropical human population over the last 2000 years. Biomass burning as evidenced from charcoal records smoothed in 200‐year windows (solid line) with mean values (dashed line) and 95% shading confidence intervals for (a) peatlands and (b) landscapes. Solid lines below each series indicate the number of sites (No.) contained in each non‐overlapping 25‐year bin and text points to significant change‐point years. (c) The reconstruction of global temperature from the PAGES 2 k Consortium showing the median value of multiple reconstruction methods (black line) and a 30‐year smoothing of the median (orange line) and the 2.5th and 97.5th percentiles (grey area) (Neukom et al. 2019). (d) The reconstruction of global δ_precip_ for precipitation (blue line), and δ_meteoric water_ for effective moisture (green line), which refers to the balance between precipitation and evaporation with 30‐year binned Δ18O composites (ensemble median of anomalies relative to mean for the last 2000 years) (Konecky et al. 2023). (e) Tropical human population (30° S and 30° N) from HYDE 3.3 (Klein Goldewijk et al. 2017). The vertical shading represents the Little Ice Age.

An increasing trend in tropical peatland burning was present in the 20th century (Figure 2). Therefore, to evaluate how fire has changed during the period 1900–2000 ce compared to the previous period (0–1900 ce), we developed maps of change in peatland burning across the tropical regions. We subtracted the mean PRS values during the 20th century (1900–2000 ce) from the values for the previous period (0–1900 ce) and also calculated the relative change by dividing this subtraction by the mean PRS values for the entire period (0–2000 ce) (Figure S10). For visualisation, the subtracted values were divided into eight groups according to data distribution (Figure S11): values greater than 50 (extremely strong positive signal), values between 15 and 50 (strong positive signal), values between 5 and 15 (positive signal), values between 0 and 5 (weak positive signal), values for 0 (no change), values between −5 and 0 (weak negative signal), values between −15 and −5 (negative signal), and values between −50 and −15 (strong negative signal).

### 2.5 PCA and Regression Analysis

Principal component analysis (PCA) was used to assess the influence of climatic variables on peatland burning using the *FactoMineR* (Lê et al. 2008) and *factoextra* packages (Kassambara and Mundt 2020). Modern climatic variables were sourced from WorldClim for the period of 1970–2000 ce (Fick and Hijmans 2017), and Global CHIRPS (The Climate Hazards group Infrared Precipitation with Stations) (Funk et al. 2015) with derived MCWD (maximum cumulative water deficit), over the period 1981–2020 (Silva Junior et al. 2019, 2021). The median PRS values (cubic root transformed to meet data normality) at each site, were used to represent the general burning status in each peat site over the last 2000 years (Figure S12). These climatic variables were selected based on their correlation (|*r*| > 0.3; range: −0.37–0.32) with median PRS values and further refined based on ecological relevance and variable independence.

Different groups, that is, sub‐region, ecosystem, elevation and human pressure types were further compared using a one‐way ANOVA test for significance in mean values. For ecosystem types, peat sites were categorised into cushion/sedge peatlands, hardwood swamps and palm swamps based on modern vegetation cover because aboveground vegetation has been shown to influence carbon storage capacity (Draper et al. 2014), recalcitrance to decomposition (Hodgkins et al. 2018) and flammability (Crawford et al. 2024). Sites with other vegetation covers were assigned to other types. There was one site (Yawi Ti, in Papua New Guinea) with no information on current vegetation. For different altitudinal types, we followed a similar classification as performed in Amazonian peatlands (Malpica‐Piñeros et al. 2024), and therefore peatlands were divided into three elevation groups: (a) lowland group below 500 m a.s.l., (b) upland group between 500 and 1500 m a.s.l., and (c) highland group above 1500 m a.s.l. Human footprint in 2000 ce was used (Williams et al. 2020) for human pressure and classified using human footprint values (range: 0–50) to differentiate between ‘wilderness’ (< 1)—representing minimal human influence, ‘intact’ (between 1 and 4) and ‘highly modified’ (> 4).

Multiple linear regression and linear mixed effect regression were further employed to assess whether significant relationships (*p* < 0.05) exist between modern climatic variables and peatland burning status using the *lme4* package (Bates et al. 2014). As well as median PRS values over the last 2000 years, three time periods, that is, 0–850 ce, 850–1900 ce and 1900–2000 ce, were also used to evaluate changes in climatic controls over time. Variables with a correlation coefficient ∣*r*∣ > 0.25 were included, and backward selection was applied to refine the models. To avoid multicollinearity, highly related variables (*|r*| > 0.8) were excluded. Model selection was carried out using the *bbmle* package (Bolker et al. 2010). Additionally, linear regression was applied for each subregion to examine the temporal changes in these three time periods.

### 2.6 Statistical Analysis

Change‐point analysis was performed on composite PRS curves to detect changes in mean and variance using ‘At Most One Change’ via the *changepoint* package (Killick and Eckley 2014). Kruskal‐Wallis rank sum test (non‐parametric test) was used to determine if there were statistically significant differences (*p* < 0.05) in 20th‐century peatland burning between sub‐regions. All statistical analyses were performed using R version 4.3.0 (R Core Team 2023).

## 3 Results

### 3.1 Peatland Burning Over Time and the Increase in the 20th Century

Higher charcoal influx indicates more intense burning levels in the wider landscape compared to peatlands throughout the fire history (Figure 2). Both burning of peatlands and the wider landscape had an overall declining trend over the last two millennia, with the decrease in the wider landscape being more pronounced than on peatlands. Elevated biomass burning in peatlands was seen before 850 ce, followed by a period of relatively stable and less burning, which was below the average for the whole period, until a recent increase that commenced in 1900 ce (Figure 2; Table S3). The composite record of landscape burning reached local peaks at 75, 500 and 800 ce, and then a continuous decrease until ~1800 ce (local minimum), with a subsequent and abrupt rise from that date towards 2000 ce.

**FIGURE 3:**
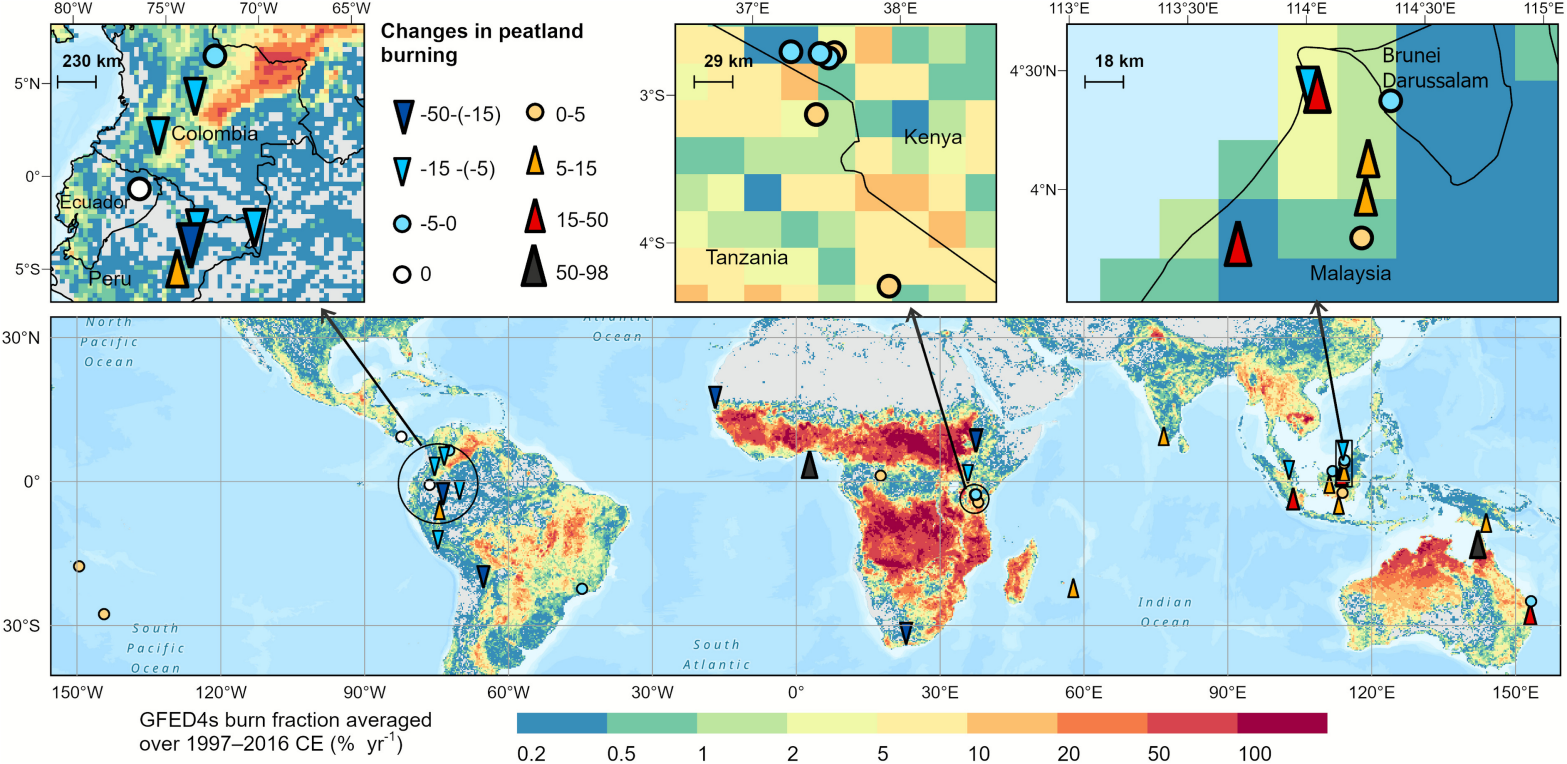
Reconstructions of changes in peatland burning between the 20th century (1900–2000 ce) and the period of 0–1900 ce. These changes are expressed as subtraction in proportional relative scaled charcoal influx between the averages in both periods. The symbols on the map point to the position of study sites, with the direction indicating increase/decrease in peat burning and the size of the symbol indicating the degree of change. Overlapping sites are zoomed in on the top panel. The background colours represent the GFED4s averaged burned fraction from 1997 to 2016 ce (Van Der Werf et al. 2017). Map lines delineate study areas and do not necessarily depict accepted national boundaries.

To illustrate the changes in peatland burning from 1900 to 2000 ce compared to the previous period (0–1900 ce), forty‐seven sites (81% of all sites) that contained available charcoal data for both periods were included (Figure 3). The upward trend was only slightly more prevalent with 23 sites showing increased peat burning and 7 sites indicating strong to extremely strong positive signals. Two sites, Oropel and Yasuní in the Neotropical region showed no change in peatland burning; however, charcoal pieces were found at ~2015 ce in Oropel. Twenty‐two sites showed a decreasing trend, with six of these sites exhibiting strong negative signals.

There were significant differences between all geographical regions in the 20th century (*p* < 0.01, Kruskal‐Wallis test). An increase in peatland burning was more pronounced in the sites located in the Australasian, Indomalayan and Oceanian regions, whereas the burning reduction was most dominant in the Neotropical and Afrotropical regions. It is worth noting that the Ahanve site from West Africa, and the Bar20 and Zurath Islet sites from northern Australia showed extremely strong positive signals. Significant differences were also found between three main ecosystem types: cushion/sedge peatlands, hardwood swamps and palm swamps (*p* < 0.01, Kruskal‐Wallis test); hardwood swamps exhibited the highest value (median ± IQR: 6.57 ± 18.40), after which cushion/sedge peatlands (median ± IQR: −0.44 ± 8.79) and palm swamps (median ± IQR: −6.76 ± 7.03). No difference was seen in elevation groupings.

### 3.2 Regional Variability in Peatland Burning

**FIGURE 4:**
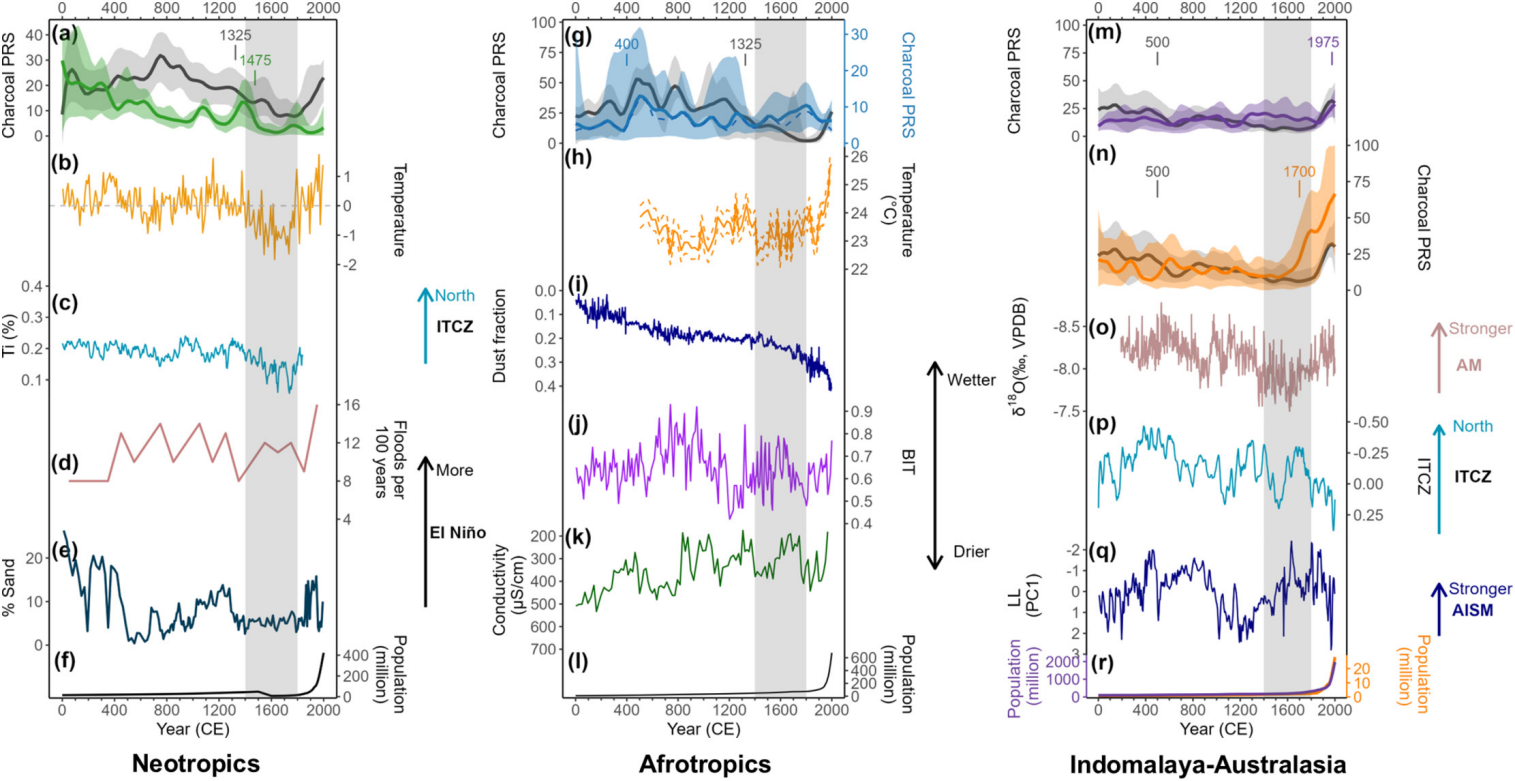
Reconstruction of biomass burning in subregions and associated climatic and human proxies over the last 2000 years. (a–f) for the Neotropical region: (a) Peatland burning (green) and landscape burning (black). Composite curves were smoothed with a 200‐year window with 95% bootstrap confidence intervals (shaded area); (b) Temperature reconstructions from three Andean δ18O ice‐core records (Thompson et al. 2006); (c) Ti (%) concentration in Cariaco Basin sediment (Haug et al. 2001); (d) El Niño events per 100 years in Laguna Pallcacocha (Mark et al. 2022); (e) Percent of sand in El Junco Crater Lake sediments (Conroy et al. 2008) and (f) Human population. (g–l) for the Afrotropical region: (g) Peatland burning (solid blue) with burning solely generated from ten East Africa sites (dashed blue) and landscape burning (black). Composite curves were smoothed with a 200‐year window with 95% bootstrap confidence intervals (shaded area); (h) TEX_86_‐inferred lake‐surface‐water temperature in Lake Tanganyika (Tierney et al. 2010); (i) Dust fraction at site GeoB9501 (Mulitza et al. 2010); (j) BIT index‐inferred precipitation from Lake Challa (Buckles et al. 2016); (k) Reconstructed lake conductivity in Lake Tanganyika (Stager et al. 2009) and (l) Human population. (m–r) for the Indomalayan and Australasian regions: Peatland burning in the Indomalayan (purple) (m) and Australasian (orange) (n) regions and combined landscape burning (black). Composite curves were smoothed with a 200‐year window with 95% bootstrap confidence intervals (shaded area); (o) Stalagmite δ18O record from Wanxiang Cave (Zhang et al. 2008); (p) The ITCZ shift index series from Klang Cave and Liang Luar Cave (Tan et al. 2019); (q) PC1 of Liang Luar Cave (Griffiths et al. 2016); (r) Human population. Human population data were derived from HYDE 3.3 (Klein Goldewijk et al. 2017). Numbers in (a), (g), (m) and (n) indicate significant change‐point years. The vertical shading represents the Little Ice Age. AM, Asian summer monsoon; AISM, Australian‐Indonesian summer monsoon.

Peatland burning showed spatiotemporal differences in four main sub‐regions with a general downward trend presenting only in the Neotropical region, but landscape burning declined for the whole period in all sub‐regions. In contrast, the burning in peatlands and landscapes during the last centuries seems to be unprecedented (Figure 4; Table S3). Due to the lack of non‐peat records in the Indomalayan‐Australasian region, we combined these two neighbouring ecoregions for wider landscape burning. Higher landscape burning in the Neotropical and Afrotropical regions was observed most of the time, whereas the Indomalayan and Australasian peatlands have experienced higher biomass burning than the broader landscapes over the past 800 years.

Trends of peatland and landscape fires in the Neotropical region mirror those in the tropics (*r* > 0.8) (Figures 2 and 4a). There was a step‐wise decrease in peatland burning over time and a significant rise around 1900 ce (Table S3); however, a decline in landscape burning only started after 800 ce and a recent sharp increase aligned with that in the tropics. Peatland burning over the entire period in the Afrotropical region remained relatively low and stable among all sub‐regions (Figure 4g), while peaks occurred at c. 500 and 1800 ce, with the first peak coinciding with high charcoal influx in landscape burning in the same region. Peatland burning in the Indomalaya and Australasia had similar levels before 1600 ce (Figure 4m,n). However, peatland burning in these two regions increased markedly during the recent 100 and 400 years, respectively. There was a drop during the increase period in Australasia, which could be biased by the decrease in the number of sites compiled (Figure S13). Elevated burning levels in the Indomalayan‐Australasian landscapes were prominent at the beginning of the Common Era and again in the last 300 years.

### 3.3 Peatland Burning Status Across Climatic Space: Influence of Sub‐Regions, Ecosystem, Elevation and Human Pressure

**FIGURE 5:**
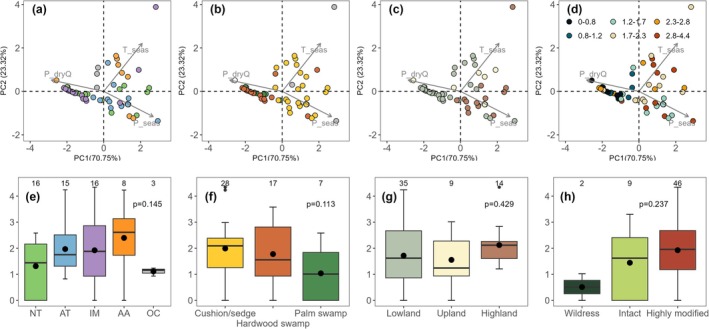
Principal component analysis (PCA) of peat sites across climatic space, with boxplots illustrating the variation in peatland burning across sub‐regions, ecosystem types, elevation groupings, and human pressure categories. In the PCA plots (a–d), the following abbreviations are used: P_dryQ—precipitation of driest quarter; P_seas—precipitation seasonality and T_seas—temperature seasonality (Fick and Hijmans 2017). Points representing individual peatland sites are coloured by (a, e) geographical regions (NT = Neotropics, AT = Afrotropics, IM = Indomalaya, AA = Australasia and OC = Oceania), (b, f) peatland ecosystem types (cushion/sedge, hardwood swamp and palm swamp with other or unknown types showing as grey or white circles), (c, g) elevation groupings (lowland, upland and highland), (d) peatland burning (median charcoal influx after proportional relative scaling and cubic root transformed) and (h) human pressure on 2000 ce grouped into wilderness, intact and highly modified (Williams et al. 2020). Boxplots (e–h) display median (thick horizontal line), mean (black point), interquartile range (box), whiskers (vertical line) and extremes of peat burning in corresponding categories with number of sites contained in each group shown on top of each box. Non‐significant differences were observed in boxplots (e–h) based on one‐way ANOVA, with *p*‐value shown in the plot.

Climatic variables are directly related to peat formation, as well as controlling peat dryness and the likelihood of ignition, thus influencing fire occurrence. To investigate this, we used the median charcoal value over the entire 2000 years to represent the burning status at each site and explored the relationship between peatland burning and climatic variables under modern environmental settings. PCA analysis suggests that modern climatic variables can generally differentiate peat burning status (Figure 5d) ‐ sites with high precipitation during the driest quarter and low seasonality in precipitation and temperature (i.e., very wet sites with relatively stable climate) do come out as being more fire‐free/−rare environments, and these sites also burn less. Conversely, most peat sites with higher charcoal values tend to occur at places where variable climate exists (high seasonality in precipitation and temperature). However, the occurrence of fires does not strictly follow certain climate patterns, as intense burning can also be observed in very wet conditions, suggesting that this process is also affected by factors beyond climate.

Geospatial attributes and human pressure could also play a role in shaping the microclimate in peatlands. Thus, we mapped geographical coverage, ecosystem and elevation types on the same PCA plot. Human pressure is not plotted, because the majority of our sites (~79%) are under moderate or intense human pressure (‘highly modified’ category) in 2000 ce. Peatlands, particularly lowland hardwood swamps and palm swamps located in the Indomalayan and Neotropical regions, share the most similar climates of less seasonal variation, whereas all palm swamps in this study are exclusively found in the Neotropics. Slightly higher charcoal values are found in hardwood swamps in Indomalaya (median: 1.67; *n* = 13) compared to palm swamps in the Neotropics (median: 1.04; *n* = 7). Another set of peatlands located in the Neotropics is high‐elevation cushion/sedge peatlands with higher seasonality (median: 1.89; *n* = 6), and this type of peatland is also seen in the Afrotropical region (median: 1.69; *n* = 10). Peatlands in Australasia are all ‘highly modified’ and mostly covered by cushion/sedge plants (median: 2.6; *n* = 6). However, none of the three geospatial attributes (geographical coverage, ecosystem and elevation types) or human pressure were statistically significantly (*p* < 0.05, ANOVA) related to burning, and any potential effects most likely exist in sub‐regions and ecosystem types (Figure 5e–h).

When considering peatland burning status based on median PRS values over the last two millennia, longitude and precipitation during the driest month emerged as the dominant drivers (Table S4). This suggests that reduced rainfall in the driest month, possibly linked to extreme droughts, intensifies peatland burning, and longitude is likely to reflect broad‐scale continental differences (including differences in human modification of the peatlands). However, treating the region as a fixed factor instead in the linear mixed effect model did not improve performance. When analysing the three different time periods, peatland burning during the 0–850 ce time period seems to have been significantly affected by precipitation seasonality, with insignificant contribution from temperature seasonality. Higher seasonality in both parameters was synchronous with elevated burning levels. During the period of 850–1900 ce, temperature seasonality explains only 7% of the variance, whereas regional variability is the main feature that largely determines the level of peatland burning in the 20th century.

### 3.4 Peatland Burning and Ecosystem Types

Although the overall difference in median PRS values representing different peatland types is not significant, palm swamps do present the lowest values, and these ecosystems occur in very wet areas. The differences between the temporal trends of different peatland types over the last 2000 years were further investigated for three main ecosystem types in our datasets (Figure S14). A decline in burning of cushion/sedge peatlands (slope = −0.003, *p* < 0.01) was observed over time, yet charcoal values in palm swamps have been much more variable than those of any other peatland type which may be due to the small number of sites. Biomass burning in the hardwood swamps showed relatively stable levels before 1800 ce but with the highest rise during the last 150 years to unprecedented levels. This is the case, even though the climate distribution for this peatland type is very similar to the palm swamp types. Change‐point analysis identified 850, 1925 and 1475 ce for cushion/sedge peatlands, hardwood swamps and palm swamps, the same as the tropics and Indomalaya and Neotropics, respectively.

## 4 Discussion

### 4.1 Peat Burning in the Tropics

#### 4.1.1 Overview

The long‐term decline in biomass burning in peatland and the wider landscape before 1800 ce aligns with trends in the Northern Hemisphere and globally (Marlon et al. 2008). A downward trend between 1800 and 1900 ce was only observed in peatland burning, which may be explained by a preference for non‐peat landscapes for agriculture, resulting in less human influence or land‐use related changes on peatland ecosystems during this period (Marlon et al. 2008; Sim et al. 2023). However, the following increase of peatland burning and also the wider landscape burning towards 2000 ce contrasts with a fairly widespread decrease in recent mid‐ to high‐latitudes peatland burning (Sim et al. 2023), and this reduction outside the tropics is thought to be linked to fire suppression policies and firefighting efforts (Mouillot and Field 2005) and landscape fragmentation (Marlon et al. 2008). The overall elevated burning was prevalent in non‐peatland ecosystems, suggesting peatland burning is buffered by the fact that they remain wetter for longer under water deficient conditions than other ecosystems.

Previous evaluation of contemporary fires has already highlighted the importance of temperature in increasing fuel flammability (Flannigan et al. 2016) and decreasing atmospheric moisture availability (Jain et al. 2022). The reconstruction of global temperatures (Neukom et al. 2019; Kaufman and Broadman 2023) suggests that during the first millennium (0–1000 ce), the climate was relatively warm and stable, contributing to the stable global hydroclimate (Konecky et al. 2023) and aligned with constant levels of peatland and landscape burning above the 2000‐year mean (Figure 2). Over the past 1000 years, the decrease in both peatland and landscape burning echoes the general cooling trend in temperatures, a period of cooling culminating in the Little Ice Age (LIA; the coldest temperature anomalies at *ca*. 1400–1800 ce (Mann et al. 2009)), and coinciding with the local minimum in both peatland and landscape burning. Condensation leading to more stable hydrological conditions is more important during cooler phases (Konecky et al. 2023), which could lower fire occurrence.

Likewise, precipitation of the driest month and the seasonality in precipitation and temperature, have also been found to control peatland burning through water deficit in peatlands during drought events or during the dry season (Hayasaka 2023). Drying in peatlands exposes belowground fuel and enhances the probability and intensity of peatland fires (Turetsky et al. 2015). Anthropogenic drying could result in a similar or even more severe risk of water deficit and subsequent fires (Page et al. 2022).

The increase in peatland burning in the 20th century may be the consequence of anthropogenic warming combined with land management practices associated with the rapid growth in human population, although certain regions may have a long history of managing and using peatlands. For instance, peatlands located in high‐elevation areas, which are prime regions for food production in all tropical countries (Potapov et al. 2022), have likely been used for grazing and other farming for a long time, such as in the Andes (Buytaert et al. 2006; Schittek et al. 2015) and in the mountains of East Africa (Githumbi et al. 2018). Peatlands on high elevations are mostly covered by cushion/sedge plants, as such plants have functional types that are adapted to high alpine conditions (Billings and Mooney 1968) and some of them are fire‐adapted (Kirkpatrick and Harding 2018). Global human population already exhibited a steady and slow increase before 1800 ce (Klein Goldewijk et al. 2017), and a significant increase has only been observed for the last 200 years (Macfarling Meure et al. 2006), suggesting that human activity was unlikely to contribute synchronous changes across the continental scale before that time. Our results suggest that temperature has controlled the general trend in peatland burning history for the tropical region over the long term, as has been suggested for other biomes. In recent centuries, however, human activities have largely contributed to regional variability in peatland burning trend.

#### 4.1.2 Sub‐Regional Analysis

Temporal trends in peatland burning differ by ecoregions. The decline in Neotropical peatland burning (Figure 4a) is possibly driven by a transition towards cooler and wetter climates over the long term, with the southward movement of the Inter‐Tropical Convergence Zone (ITCZ) (Haug et al. 2001). Additionally, burning might be associated with El Niño–Southern Oscillation (ENSO) activity on interannual timescales, because El Niño can promote a reduction in precipitation in the Amazon Basin and northeastern South America (Cai et al. 2020). El Niño activity was relatively intense before the LIA with most peaks in peatland fire happening before this date; while relatively fewer El Niño events happened during the LIA cool period (Conroy et al. 2008; Mark et al. 2022) (Figure 4d,e). This, combined with lower temperatures and the highest rainfall resulting from the southernmost position of ITCZ over the Common Era (Haug et al. 2001; Thompson et al. 2006) could have contributed to the local minimum in peatland burning during the LIA. Additionally, the human population collapsed after 1492 ce due to disease epidemics brought by European arrivals (Denevan 1992; Koch et al. 2019), and this could also have contributed to a much reduced peatland burning during the LIA. This reduction was also observed for the wider landscape (Feldpausch et al. 2022).

Peatlands in tropical Africa experience higher precipitation or temperature seasonality than most Neotropical peatlands and ~63% of the sites in our compilation are from high elevations in this region (Figure 5). Peatland burning in the Afrotropical region mirrors the burning patterns solely generated from east Africa (*n* = 10 out of 15) (Figure 4g) although climatic variations differ for specific regions in Africa. For example, precipitation in east Africa increased during 0–1000 ce (Figure 4j,k) (Stager et al. 2009; Buckles et al. 2016), while at the same time decreased in west Africa (Figure 4i) (Mulitza et al. 2010). A high‐resolution temperature reconstruction from Lake Tanganyika in east Africa (Tierney et al. 2010) showed a relatively warm period between 500 and 700 ce, but depression for the LIA was not clearly evident (Figure 4h). This warm period happened in parallel with increased fire episodes in peatlands and landscapes and is likely to be linked to a widespread drought event mainly expressed in the Northern Hemisphere as this aridification weakened as it approached the equator (Nash et al. 2016). The subsequent interval of decreasing burning in both peatlands and the wider landscapes until 1400 ce resulted from wetter conditions in east Africa (Stager et al. 2009; Buckles et al. 2016). Landscape burning for the studied period is also driven by sites in east Africa (*n* = 8 out of 11). During the LIA, the cool climate and intermittent dry years, as well as the loss of human labour in Africa from the slave trade (Lovejoy 1989; Eltis 2007) could have reduced the capacity for widespread landscape burning. However, this decline contrasts with a continuous increase in peatland burning during the same period, and the disparity during the LIA is likely a reflection of differing land management practices—that is, high mountain ecosystems have likely been targeted for agricultural practices, or few available records in landscape burning in this region.

The Indomalayan peatlands are mostly lowland sites that are very similar to lowland Neotropical sites in terms of climate, whereas the climatic conditions for Australasian peatlands are more similar to those in tropical Africa (Figure 5). The Indomalayan‐Australasian region records a similar feature between peatland and wider landscape burning, characterised by a high increase in recent times (Figure 4m,n). The general decline in landscape burning until the LIA coincides with that of other tropical regions and is likely related to the decline in global temperatures. The Asian summer monsoon and Australian‐Indonesian summer monsoon (Zhang et al. 2008; Griffiths et al. 2016) have both experienced large variations over time controlled by the movement of ITCZ (Tan et al. 2019); however, only minor changes were reflected in peat burning for the entire period, while human drainage combined with droughts (Cook et al. 2010; Tibby et al. 2018) in the recent centuries has led to burning levels that exceed any previous burning. This sharp rise was also found in landscape burning, coinciding with increases in human population (Goldewijk 2005; Klein Goldewijk et al. 2017) and related landscape conversion (Cole et al. 2019; Page et al. 2022).

### 4.2 Peatland Fires and Possible Human Interaction Within Ecoregions and Ecosystem Types

Apart from climatic controls, our results also suggest a link between higher human pressures and more peatland burning, especially in recent centuries. For example, human population density in the Indomalayan region has reached 249 inhabitants km^−2^ on average in 2000 ce, at least seven times more than all the other sub‐regions (27, 33 and 5 inhabitants km^−2^ for the Neotropics, Afrotropics and Australasia, respectively) (Klein Goldewijk et al. 2017). The higher population growth rate has persisted over time and so the Indomalayan region continues to be the most populous region (Klein Goldewijk et al. 2017) and a region where peatland has been more widely used for palm and acacia plantations as well as smallholder agriculture (Page et al. 2022). This coincides with higher levels of peat burning in Indomalaya. The Australasian region (mainly located in northern and eastern Australia) has experienced the highest peat burning increase over the past 300 years, which corresponds to a time of wetter climate in eastern Australia (Tibby et al. 2018). This increase in peatland burning of Australasia aligns with the wider landscape burning trends obtained from this study and from a study evaluating more than a thousand samples in this region (Mooney et al. 2011). Although Australasia has the lowest population density among all the sub‐regions, higher levels of burning in Australasia started at an earlier date, ~1600 ce, which may be related to changes in fire practices by Aboriginal people, whilst the later increase over the last 150 years is likely to be related to European colonisation since ~1788 ce (Gergis et al. 2010; Mariani et al. 2024) combined with more frequent ENSO events (Mark et al. 2022).

Human effects in Southeast Asian hardwood swamps in the 20th century are the most pronounced, including the use of fire as a low‐cost tool for agricultural expansion, so these peatlands have been more extensively deforested and drained compared to all the other lowland tropical regions (e.g., Koh et al. (2011); Miettinen et al. (2012)). As a result, significant carbon emission from severe peat loss in Indonesia has been identified in the global carbon budget (Randerson et al. 2015). Burning in palm swamps from the Neotropical region and hardwood forests in Africa (Site: Ekolongouma) and French Polynesia (Site: Ra'irua) is low at present. This is most likely due to the lack of human ignition sources and low fire ignition probability in naturally permanently waterlogged peatlands in these regions. Large‐scale land development and conversion have only recently begun to be planned or are still in the early exploration stages (Roucoux et al. 2017; Dargie et al. 2019).

### 4.3 Limitations and Uncertainties

Our datasets provide the first compilation of tropical peatland burning and reveal a recent, pronounced rise in burning levels in the Indomalayan and Australasian ecoregions. The fate of peatlands in the Neotropical and Afrotropical regions remains uncertain, primarily due to the uncertainties regarding ongoing local threats and the existence and effectiveness of legal protections. Our results are limited by the number and locations of sites from which peat records exist, especially compared to mid‐ to high‐latitude peatlands (Sim et al. 2023). There is far less research in some regions; for instance, the central Congo Basin represents the largest known tropical peat complex (Crezee et al. 2022), but there is only one existing charcoal record that could be included in this study. Notably, our compilation excluded micro‐charcoal records, which are commonly counted in palynological analyses and generally reflect larger‐scale fires or regional fire patterns (Hope 2009; Kelly et al. 2017). More effectively differentiating between charcoal size classes could convey different aspects of fire history. Additionally, establishing a standard protocol may help clarify the specific questions addressed by each charcoal fraction (Vachula 2019).

The development and type of peatland settings vary across sites, and the local factors, such as microclimate, topography, and hydrology can also influence the accumulation of charcoal in the peat profile (Cobb et al. 2017; Morris et al. 2018). Most of the palaeoecological peat research tends to collect cores from intact peatlands and/or the deepest parts of a given peatland; while this sampling bias ensures robust recovery of past proxies spanning the longest time, it could also introduce bias in the interpretation of fire regimes, as peat fires are more likely to be suppressed in the central region of a peatland.

The uncertainty of the age‐depth models could be greatly reduced by increasing the number of dating points covering the period of interest. Our compilation has only included records containing a minimum of two dating points (Figure S15). Despite this, the general decline over time and the recent increase in burning during the 20th century in the tropics are robust findings. High‐resolution dating is cost‐intensive, especially for the very deep peat cores, and factors such as fluvial intrusion and root penetration could result in ‘inconsistent’ ages within the peat profile (Hapsari et al. 2017; Ruwaimana et al. 2024). Nonetheless, the reliability of our results could be greatly enhanced by more precise age controls and better‐represented peat geographical areas.

Additionally, there remains the fundamental limitation of interpreting the potential drivers regarding the contribution from climate variability and human effects, especially for the recent two millennia, when human activities have intensified. The distinction between these two drivers only works well when one clearly overrides the other, for example, during periods with minimal or an absence of anthropogenic activities or during periods of fire with unfavourable climatic conditions.

## 5 Conclusion

Our study highlights that climate variables related to the length and severity of dry periods could contribute to intense peatland burning or more fire‐prone conditions over time and space. The recent increase in peatland burning in the 20th century has significant regional variability and is mainly observed in the Indomalayan and Australasian regions, highlighting the importance of human activities in these regions. Relatively under‐disturbed peatlands in the Neotropical and Afrotropical regions have experienced less large‐scale effects from human activities due to their relative inaccessibility (Cole et al. 2022). However, pressure from increased population density will continue and with it, a likely expansion of commercial agriculture and infrastructure in these peat‐extensive areas (Roucoux et al. 2017; Dargie et al. 2019). Therefore, burning of these peatlands could mirror the same fate as the peatlands in Indomalaya and Australasia.

The comparison between peatland and landscape burning highlights the natural protective characteristics of peatland ecosystems that reduce the likelihood of fires. However, once peatland dries, it may be subject to higher fire risks than any other biome, especially for those that have previously experienced minimal fire disturbance. To avoid large carbon emissions that contribute to global warming and the associated effects of peatland fires (Yule 2010; Page et al. 2022), the protection of these carbon‐dense ecosystems is needed under a warmer future (Masson‐Delmotte et al. 2021). A reduction in tropical peatland burning could be achieved through peatland conservation, and promoting sustainable resource management and ecosystem restoration, but this requires the collaboration of multiple groups, and has to be carried out at sufficiently large scale (Harrison et al. 2020; Girkin et al. 2023; Hooijer et al. 2024).

## Author Contributions

**Yuwan Wang:** data curation, formal analysis, methodology, project administration, resources, visualization, writing – original draft. **Ted R. Feldpausch:** conceptualization, resources, supervision, writing – review and editing. **Graeme T. Swindles:** conceptualization, data curation, project administration, writing – review and editing. **Patrick Moss:** resources, supervision, writing – review and editing. **Hamish A. McGowan:** supervision, writing – review and editing. **Thomas G. Sim:** data curation, methodology, project administration, writing – review and editing. **Paul J. Morris:** project administration, writing – review and editing. **Adam Benfield:** resources, writing – review and editing. **Colin Courtney‐Mustaphi:** resources, writing – review and editing. **David Wahl:** resources, writing – review and editing. **Encarni Montoya:** resources, writing – review and editing. **Esther Githumbi:** resources, writing – review and editing. **Eurídice N. Honorio Coronado:** resources, writing – review and editing. **Femke Augustijns:** resources, writing – review and editing. **Gert Verstraeten:** resources, writing – review and editing. **Jess O' Donnell (Roe):** resources, writing – review and editing. **John Tibby:** resources, writing – review and editing. **Juan C. Benavides:** resources, writing – review and editing. **K. Anggi Hapsari:** resources, writing – review and editing. **Karsten Schittek:** resources, writing – review and editing. **Khairun Nisha Mohamed Ramdzan:** resources, writing – review and editing. **Kunshan Bao:** resources, writing – review and editing. **Lydia E. S. Cole:** resources, writing – review and editing. **Lysanna Anderson:** resources, writing – review and editing. **Mariusz Gałka:** resources, writing – review and editing. **Orijemie Emuobosa Akpo:** resources, writing – review and editing. **Paul Strobel:** resources, writing – review and editing. **Prabhakaran Ramya Bala:** resources, writing – review and editing. **René Dommain:** resources, writing – review and editing. **Rob Marchant:** resources, writing – review and editing. **Raman Sukumar:** resources, writing – review and editing. **Sakonvan Chawchai:** resources, writing – review and editing. **Sarath Pullyottum Kavil:** resources, writing – review and editing. **Scott Mooney:** resources, writing – review and editing. **Thomas J. Kelly:** resources, writing – review and editing. **Yang Gao:** resources, writing – review and editing. **Apostolos Voulgarakis:** writing – review and editing. **Arnoud Boom:** writing – review and editing. **Chantelle Burton:** writing – review and editing. **Juan Carlos Berrio:** writing – review and editing. **Kelly Ribeiro:** writing – review and editing. **Liana O. Anderson:** writing – review and editing. **Mark Hardiman:** writing – review and editing. **Molly Spater:** writing – review and editing. **Susan E. Page:** writing – review and editing. **Angela V. Gallego‐Sala:** conceptualization, project administration, resources, supervision, writing – review and editing.

## Funding

This work was supported by UK Natural Environment Research Council (NE/N011570/1, NE/R017980/1, NE/W001691/1). NERC Radiocarbon Dating Facility, allocation number (2353.0321). Global Challenges Research Fund (UKRI, NE/T010401/1). Burnett Mary Regional Group. NERC Radiocarbon Dating Facility (1565.0411). Beatriu de Pinós—Marie Curie COFUND fellowship (2014 BP‐B 00094). Australian Research Council Discovery Project (DP150103875). Australian Research Council Linkage Project (LP0990124). Spanish Ministry of Economy and Competitivity (IJCI‐2015‐24273). National Council for Scientific and Technological Development (314473/2020‐3, 409531/2021‐9). NERC Knowledge Exchange Fellowship (NE/V018760/2). Fund for Scientific Research—Flanders (11D7520N). U.S. Geological Survey Land Change Science Program. VLIR‐UOS, IUC project AMU (ET2017IUC035A101). PhD scholarship via the QUEX Institute (The University of Queensland and The University of Exeter). European Research Council, European Union's Horizon 2020 research and innovation programme (865403). DST‐INSPIRE Faculty Award, Department of Science and Technology, Government of India (IFA‐20‐EAS‐86). Fundação de Amparo à Pesquisa do Estado de São Paulo (FAPESP‐2020/16457‐3). Spanish Ministry of Science and Innovation (PID2022‐138059NB‐I00). Leverhulme Trust (RPG‐2021‐354).

## Conflicts of Interest

The authors declare no conflicts of interest.

## Supporting information


**Data S1:** Supporting Information.

## Data Availability

The data that support the findings of this study are openly available in figshare at https://doi.org/10.6084/m9.figshare.30061531. The data for Lago Paixban are also available from U.S. Geological Survey data release (https://doi.org/10.5066/p15ibcso).
