# Supplementary material for: Unprecedented Burning in Tropical Peatlands During the 20th Century Compared to the Previous Two Millennia
Source: Glob Chang Biol. 2026 Mar 17;32(3):e70717. doi: 10.1111/gcb.70717 (PMC12993805; doi:10.1111/gcb.70717)
Supplement: Supplementary file 1 — Data S1: Supporting Information. [file GCB-32-e70717-s001.docx]

## Supplementary Information for

**Unprecedented burning in tropical peatlands during the 20*th* century compared to the previous two millennia**

**Authors:** Yuwan Wang^1,2,3*^, Ted R. Feldpausch^1^, Graeme T. Swindles^4,5^, Patrick Moss^2,6^, Hamish A. McGowan^7^, Thomas G. Sim^8^, Paul J. Morris^9^, Adam Benfield^10,11^, Colin Courtney-Mustaphi^12,13,14,15^, David Wahl^16^, Encarni Montoya^17^, Esther Githumbi^18^, Eurídice N. Honorio Coronado^19^, Femke Augustijns^20^, Gert Verstraeten^20^, Jess O'Donnell (Roe)^21^, John Tibby^22^, Juan C. Benavides^23^, K. Anggi Hapsari^24^, Karsten Schittek^25^, Khairun Nisha Mohamed Ramdzan^26^, Kunshan Bao^27^, Lydia E. S. Cole^28^, Lysanna Anderson^16^, Mariusz Gałka^29^, Orijemie Emuobosa Akpo^30^, Paul Strobel^31^, Prabhakaran Ramya Bala^32^, René Dommain^33,34^, Rob Marchant^13,35^, Raman Sukumar^36^, Sakonvan Chawchai^37^, Sarath Pullyottum Kavil^38^, Scott Mooney^21^, Thomas J. Kelly^39^, Yang Gao^40^, Apostolos Voulgarakis^41,42^, Arnoud Boom^43^, Chantelle Burton^44^, Juan Carlos Berrio^43^, Kelly Ribeiro^45^, Liana O. Anderson^46^, Mark Hardiman^47^, Molly Spater^48^, Susan E. Page^43^, Angela Gallego-Sala^1*^

1. Department of Geography, University of Exeter, Exeter, UK
2. School of the Environment, University of Queensland, Brisbane, Australia
3. State Key Laboratory of Lithospheric and Environmental Coevolution, Institute of Geology and Geophysics, Chinese Academy of Sciences, Beijing, China
4. Geography, School of Natural and Built Environment, Queen’s University Belfast, Belfast, UK
5. Ottawa-Carleton Geoscience Centre and Department of Earth Sciences, Carleton University, Ottawa, ON, Canada
6. School of Earth & Atmospheric Sciences, Queensland University of Technology, Brisbane, Australia
7. Weather and Climate Science Research Alliance, The University of Queensland, Brisbane, Australia
8. Forest Research, Northern Research Station, Roslin, Midlothian, UK
9. School of Geography, University of Leeds, Leeds, UK
10. Department of Earth and Environment, Franklin and Marshall College, Lancaster, PA 17604
11. Department of Geological and Environmental Sciences, Appalachian State University, Boone, NC, USA
12. Geoecology, Department of Environmental Sciences, University of Basel, Klingelbergstrasse 27, 4056, Basel, Switzerland
13. York Institute for Tropical Ecosystems, Department of Geography and Environment, University of York, York, UK
14. Center for Water Infrastructure and Sustainable Energy (WISE) Futures, Nelson Mandela African Institution of Science and Technology, Arusha, Tanzania
15. Knowledge Core LLC, Basel, Basel-Stadt, Switzerland
16. U.S. Geological Survey, Geology, Minerals, Energy, and Geophysics Science Center 350 N. Akron Rd. Moffett Field, CA 94035, USA
17. Geosciences Barcelona, CSIC, c/ LLuis Solé i Sabaris s/n, Barcelona 08028, Spain
18. Institute of Soil Science and Site Ecology, TU Dresden, Piennerstraße 19, 01737 Tharandt, Germany
19. Royal Botanic Gardens, Kew, Richmond, London, TW9 3AE, UK
20. KU Leuven, Department of Earth and Environmental Sciences, Division of Geography and Tourism. Celestijnenlaan 200E, 3001 Leuven, Belgium
21. School of Biological, Earth & Environmental Sciences, The University of New South Wales, NSW 2052 Australia
22. Geography, Environment and Population, University of Adelaide, North Terrace, Adelaide, South Australia, 5005
23. Department of Ecology and Territory, Pontificia Universidad Javeriana, Bogotá, Colombia
24. Albrecht-von-Haller Institute, University of Goettingen, Untere Karspuele 2, 37073 Goettingen
25. Institute of Geography Education, University of Cologne, Gronewaldstr. 2, 50931 Köln, Germany
26. Earth Observatory of Singapore, Nanyang Technological University, Singapore
27. School of Geographic Science, South China Normal University, Guangzhou 510631, China
28. School of Geography & Sustainable Development, University of St Andrews, Irvine Building, North Street, St Andrews, Fife, Scotland KY16 9AL
29. University of Lodz, Faculty of Biology and Environmental Protection, Department of Biogeography, Paleoecology and Nature Conservation, Banacha 1/3, 90-237 Łodz, Poland
30. Department of Archaeology and Anthropology, University of Ibadan, Ibadan, Nigeria
31. Department of Physical Geography, Friedrich Schiller University Jena, Loebdergraben 32, 07743 Jena, Germany
32. National Institute of Advanced Studies, Indian Institute of Science campus, Bengaluru 560012, India
33. University of Potsdam, Institute of Geosciences, Karl-Liebknecht-Str. 24-25, 14476 Potsdam-Golm, Germany
34. Human Origins Program, National Museum of Natural History, Smithsonian Institution, Washington, DC 20013, USA
35. Faculty of Environment and Resource Studies, Mahidol University, Phutthamonthon Sai 4 Road, Nakhon Pathom, 73170, Thailand
36. Centre for Ecological Sciences, Indian Institute of Science, Bangalore 560012, India
37. Department of Geology, Faculty of Science, Chulalongkorn University, Bangkok 10330 Thailand
38. Department of Geological Sciences, Stockholm University, Stockholm, Sweden
39. School of Geography, Queen Mary University of London, Mile End Rd, Bethnal Green, London E1 4NS
40. School of Karst, Guizhou Normal University/State Engineering Technology Institute for Karst Desertification Control, Guiyang 550001, China
41. Leverhulme Centre for Wildfires, Environment and Society, Department of Physics, Imperial College London, London, UK
42. School of Chemical and Environmental Engineering, Technical University of Crete, Greece
43. School of Geography, Geology and the Environment, University of Leicester, University Road, LE1 7RH Leicester, UK
44. Met Office Hadley Centre, Met Office, Exeter, UK
45. National Institute for Space Research (INPE), Brazil
46. National Center for Monitoring and Early Warning of Natural Disasters (CEMADEN), São José dos Campos, Brazil
47. School of the Environment and Life Sciences, University of Portsmouth, Lion Terrace, Buckingham Building, Portsmouth, PO1 3HE, UK
48. Department of Geography & Planning, University of Liverpool, Liverpool L69 7ZT, UK

**This file contains:**

**Supplementary Figures 1-15**

**Supplementary Tables 1- 4**


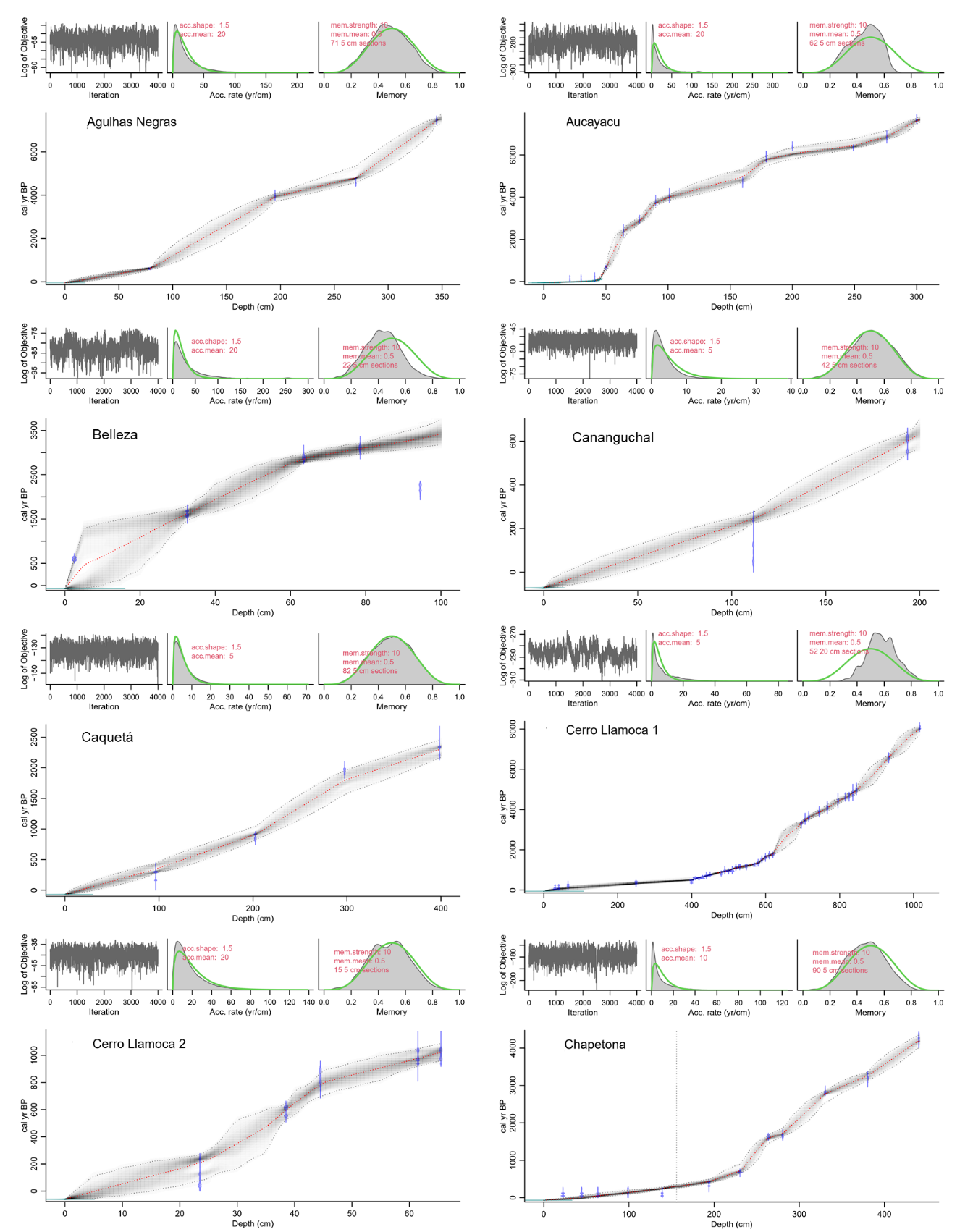


**Figure S1** Age-depth models of Agulhas Negras, Aucayacu, Belleza, Cananguchal, Caquetá, Cerro Llamoca 1 (peatland), Cerro Llamoca 2 (peat hillock) and Chapetona. All calibrated age control points show as transparent blue in the main plot. Median age for each depth (red curve) is the 'best' model and grey stippled lines show 95% confidence intervals. The prior (green curves) and posterior (grey histograms) information for the accumulation rate and memory are listed in the upper panel.


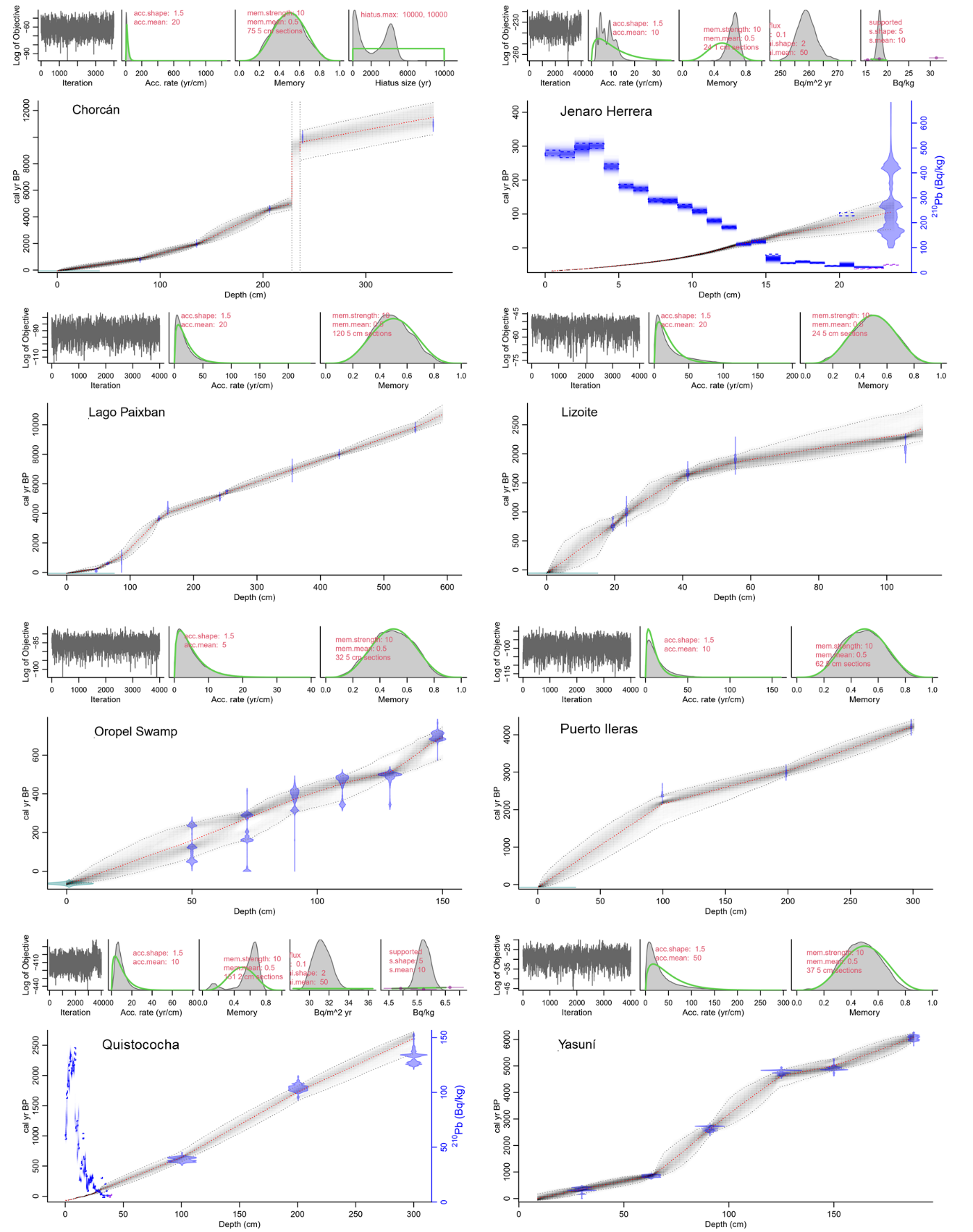


**Figure S2** Age-depth models of Chorcán, Jenaro Herrera, Lago Paixban, Lizoite, Oropel, Puerto lleras, Quistococha and Yasuní. All calibrated age control points show as transparent blue in the main plot. Median age for each depth (red curve) is the 'best' model and grey stippled lines show 95% confidence intervals. The prior (green curves) and posterior (grey histograms) information for the accumulation rate and memory are listed in the upper panel.


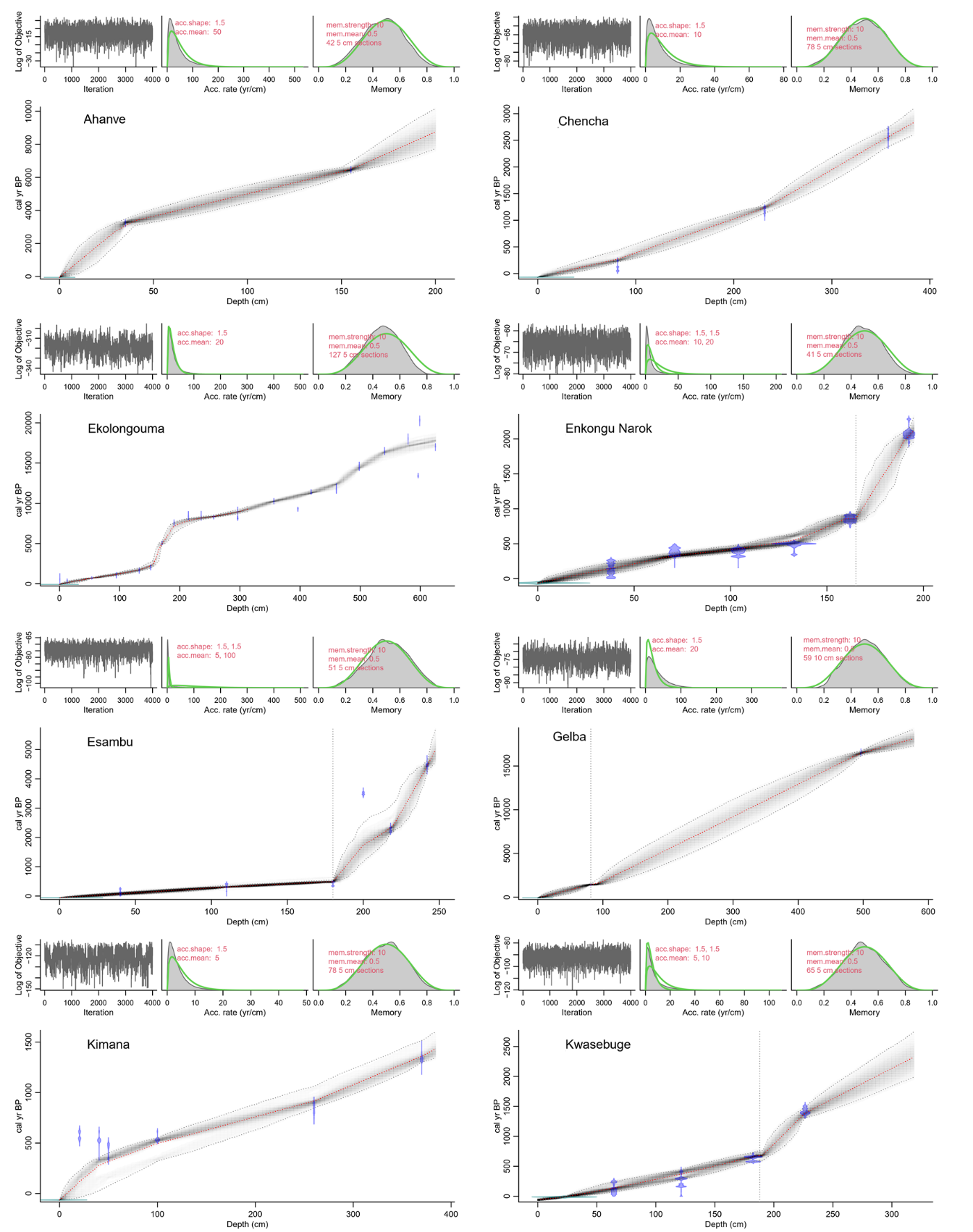


**Figure S3** Age-depth models of Ahanve, Chencha, Ekolongouma, Enkongu Narok, Esambu, Gelba, Kimana and Kwasebuge. All calibrated age control points show as transparent blue in the main plot. Median age for each depth (red curve) is the 'best' model and grey stippled lines show 95% confidence intervals. The prior (green curves) and posterior (grey histograms) information for the accumulation rate and memory are listed in the upper panel.


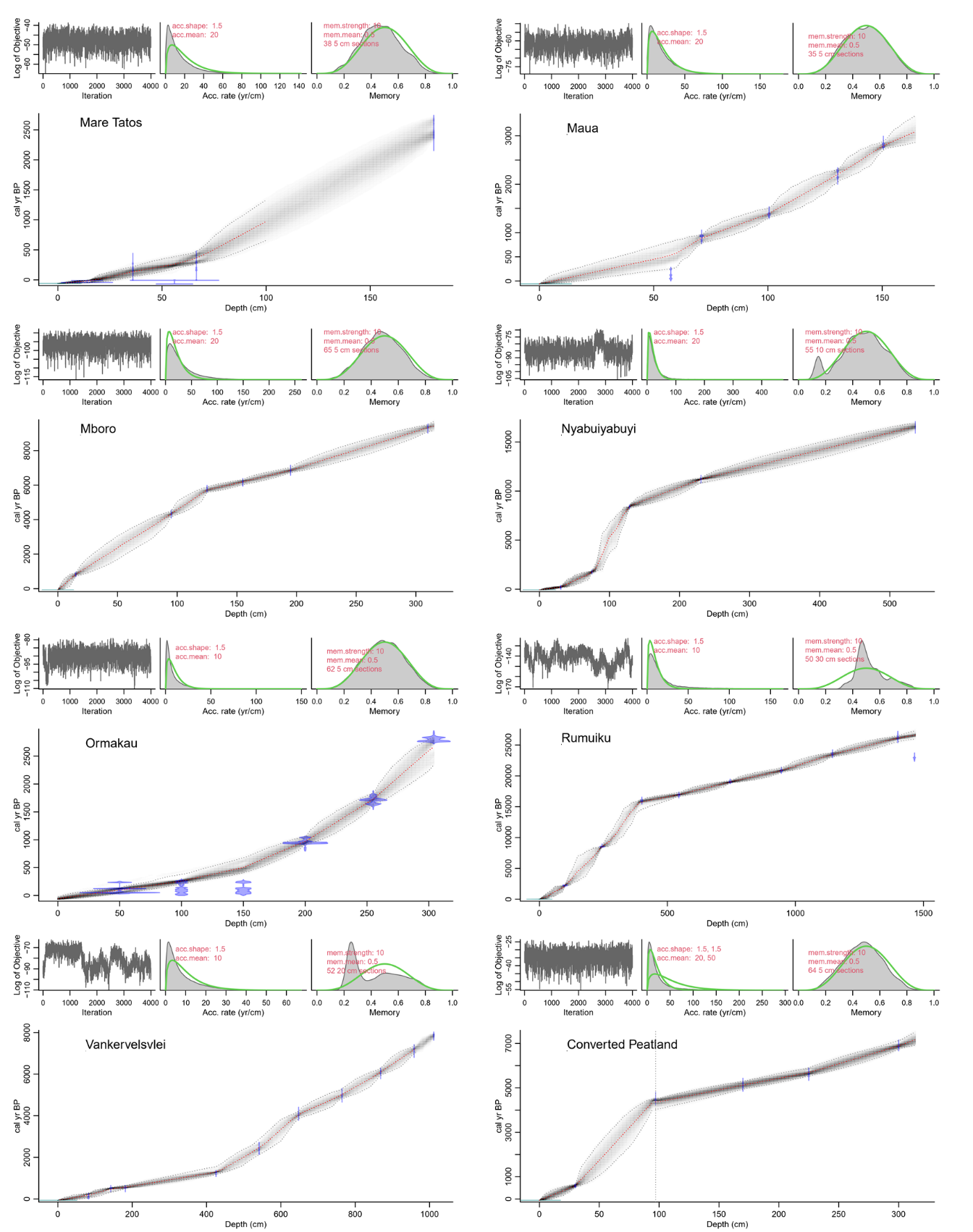


**Figure S4** Age-depth models of Mare Tatos, Maua, Mboro, Nyabuiyabuyi, Ormakau, Rumuiku, Vankervelsvlei and Converted Peatland. All calibrated age control points show as transparent blue in the main plot. Median age for each depth (red curve) is the 'best' model and grey stippled lines show 95% confidence intervals. The prior (green curves) and posterior (grey histograms) information for the accumulation rate and memory are listed in the upper panel.


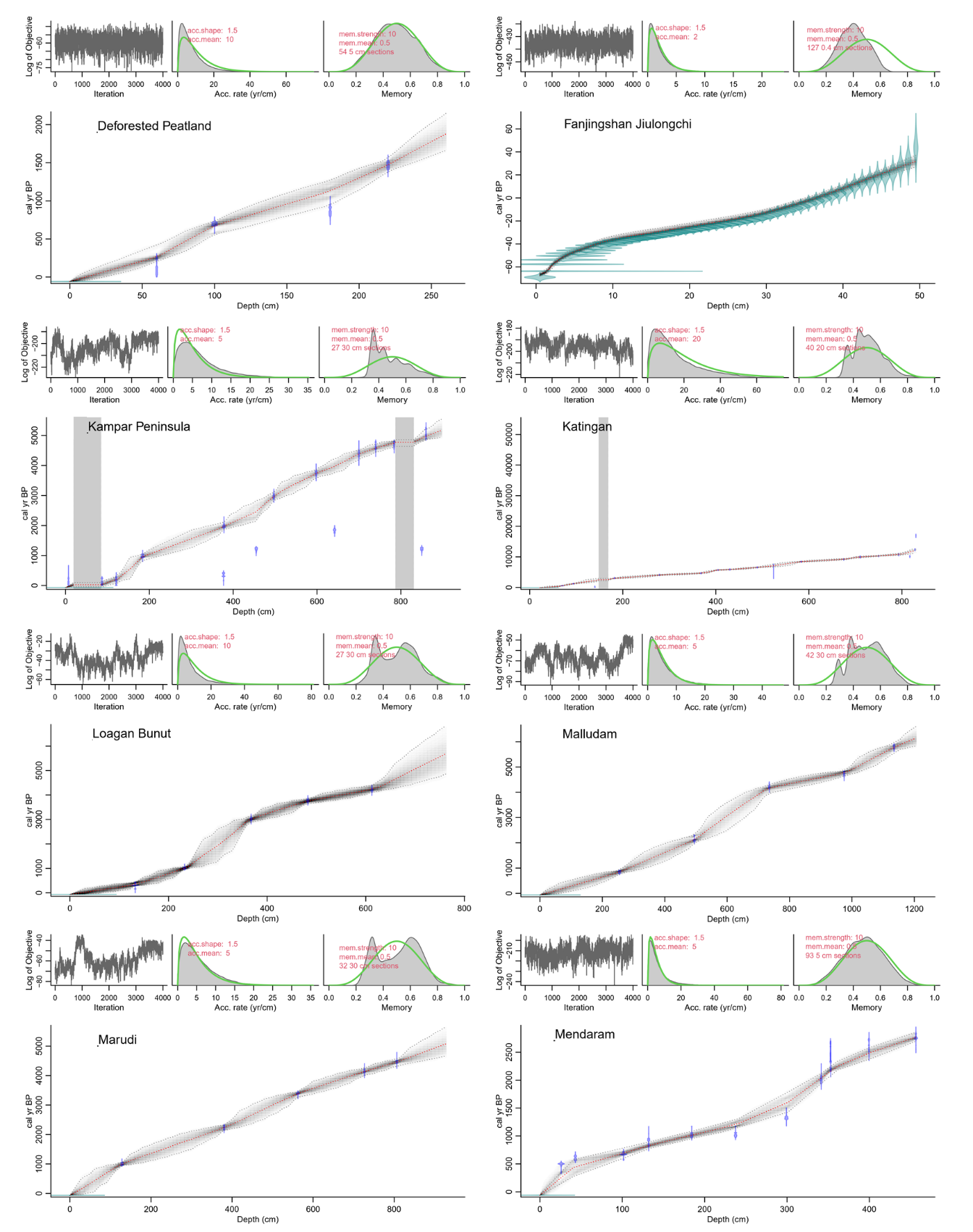


**Figure S5** Age-depth models of Deforested Peatland, Fanjingshan Jiulongchi, Kampar Peninsula, Katingan, Loagan Bunut, Malludam, Marudi and Mendaram. All calibrated age control points show as transparent blue in the main plot. Median age for each depth (red curve) is the 'best' model and grey stippled lines show 95% confidence intervals. The prior (green curves) and posterior (grey histograms) information for the accumulation rate and memory are listed in the upper panel.


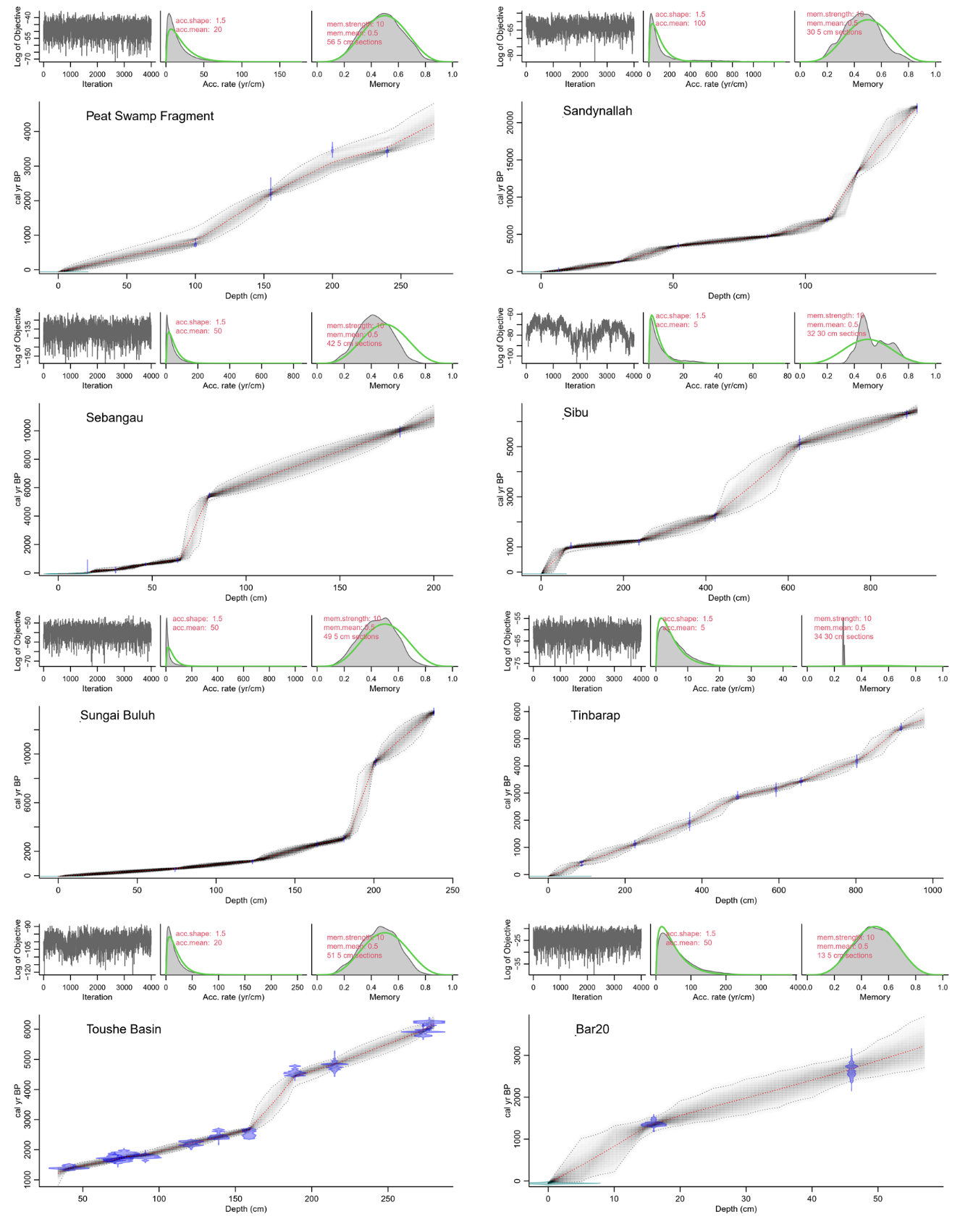


**Figure S6** Age-depth models of Peat Swamp Fragment, Sandynallah, Sebangau, Sibu, Sungai Buluh, Tinbarap, Toushe Basin and Bar20. All calibrated age control points show as transparent blue in the main plot. Median age for each depth (red curve) is the 'best' model and grey stippled lines show 95% confidence intervals. The prior (green curves) and posterior (grey histograms) information for the accumulation rate and memory are listed in the upper panel.


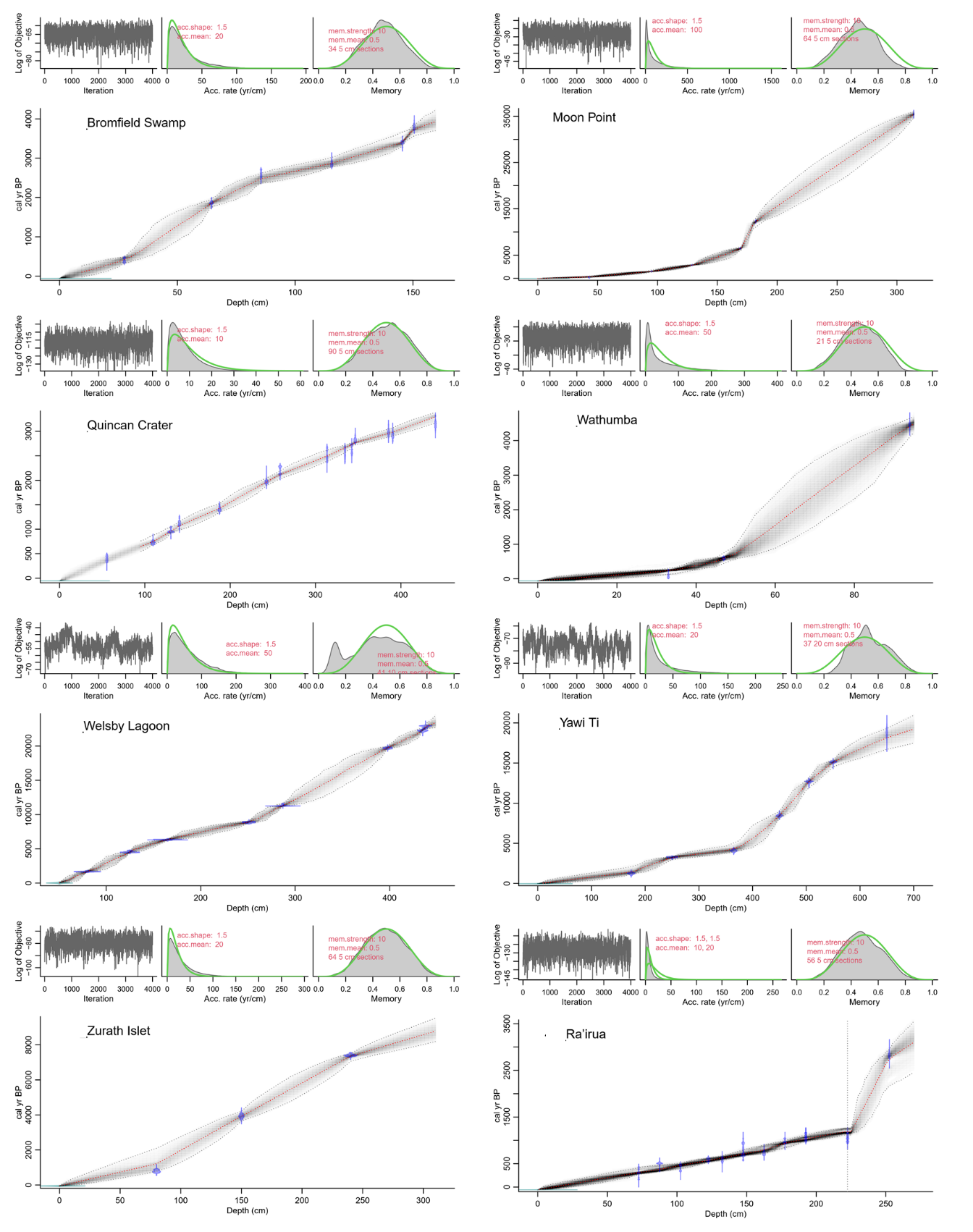


**Figure S7** Age-depth models of Bromfield Swamp, Moon Point, Quincan Crater, Wathumba, Welsby Lagoon, Yawi Ti, Zurath Islet and Ra’irua. All calibrated age control points show as transparent blue in the main plot. Median age for each depth (red curve) is the 'best' model and grey stippled lines show 95% confidence intervals. The prior (green curves) and posterior (grey histograms) information for the accumulation rate and memory are listed in the upper panel.


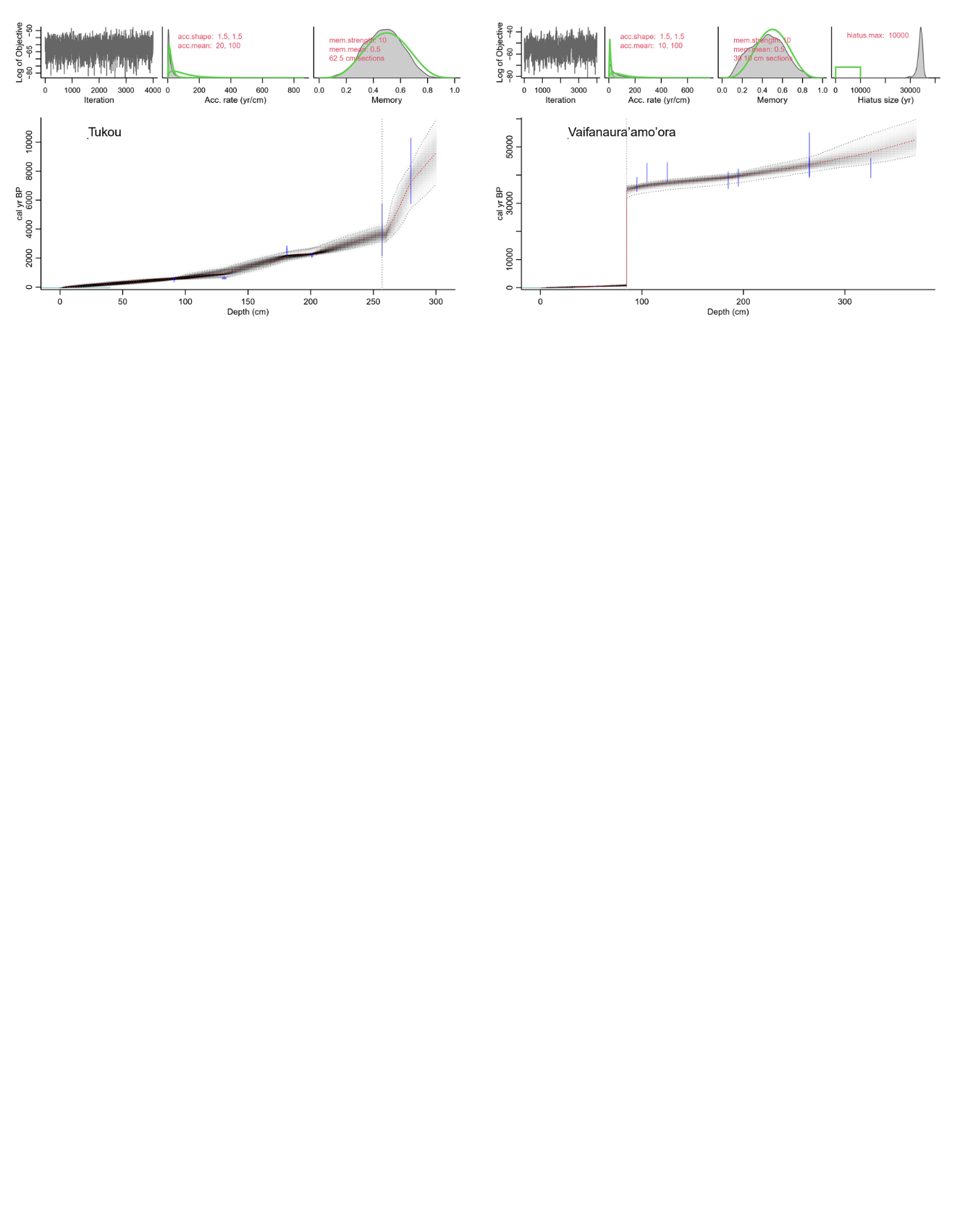


**Figure S8** Age-depth models of Tukou and Vaifanaura’amo’ora. All calibrated age control points show as transparent blue in the main plot. Median age for each depth (red curve) is the 'best' model and grey stippled lines show 95% confidence intervals. The prior (green curves) and posterior (grey histograms) information for the accumulation rate and memory are listed in the upper panel.


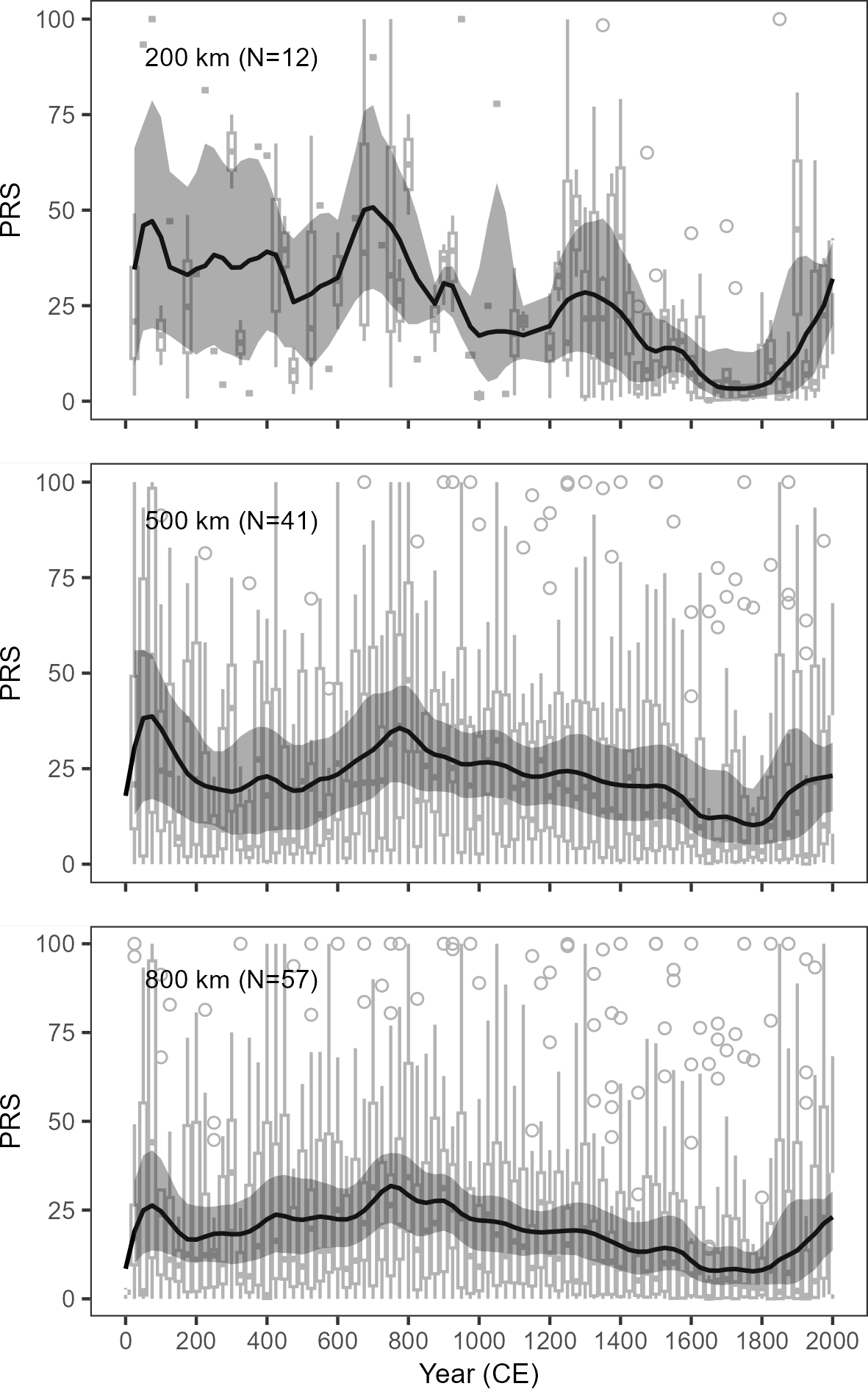


**Figure S9** Charcoal PRS values indicating non-peatland landscape burning in the Neotropical region within 200-, 500-, and 800-km buffers of peat sites.

**
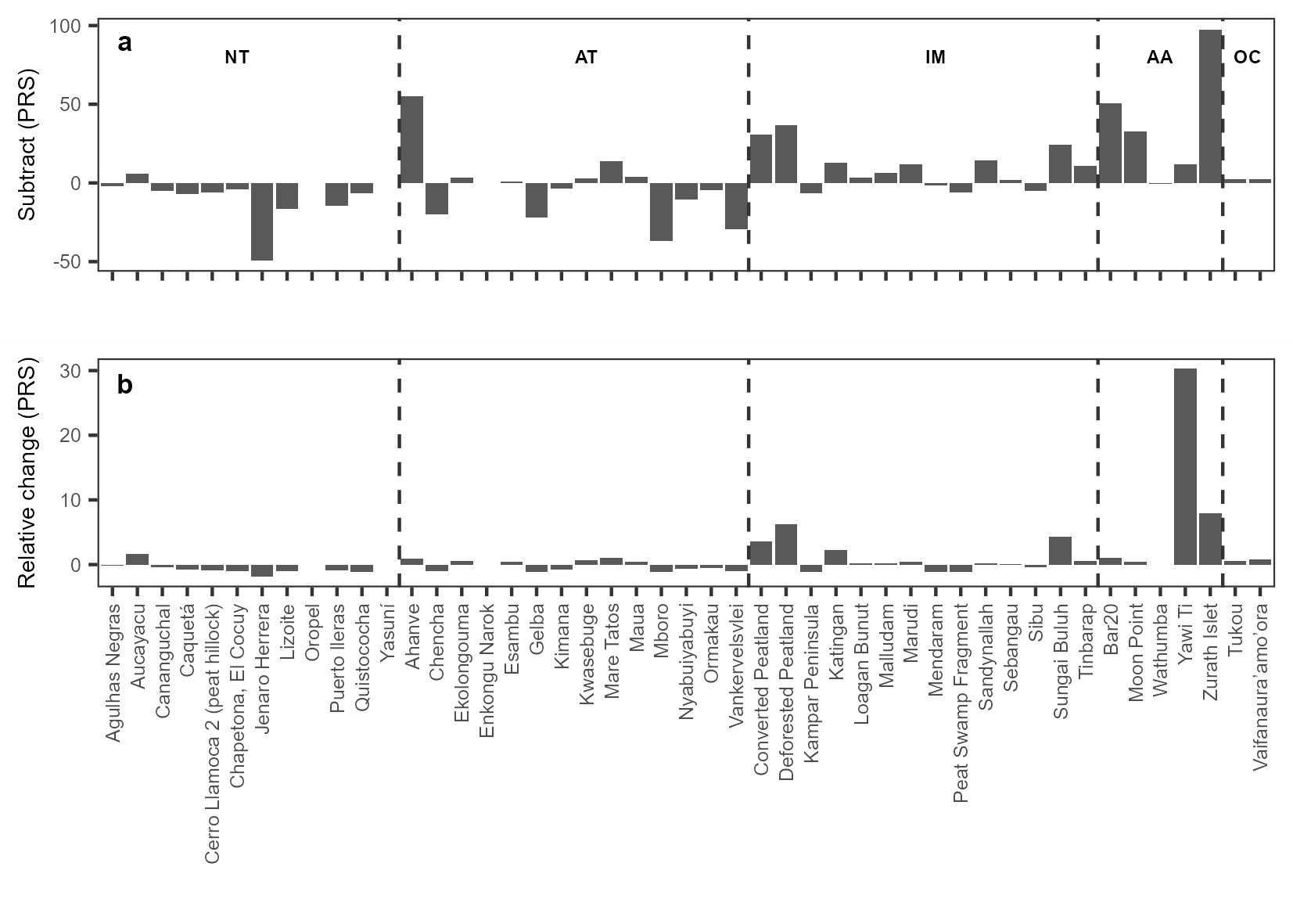
**

**Figure S10** Changes in peat burning in the period of 1900-2000 CE compared to the previous period (0-1900 CE) in sub-regions. (a) subtraction (Mean_1900-2000_-Mean_0-1900_) and (b) relative change ((Mean_1900-2000_-Mean_0-1900_)/Mean_0-2000_) based on charcoal PRS values. NT=Neotropics, AT=Afrotropics, IM=Indomalaya, AA=Australasia and OC=Oceania.


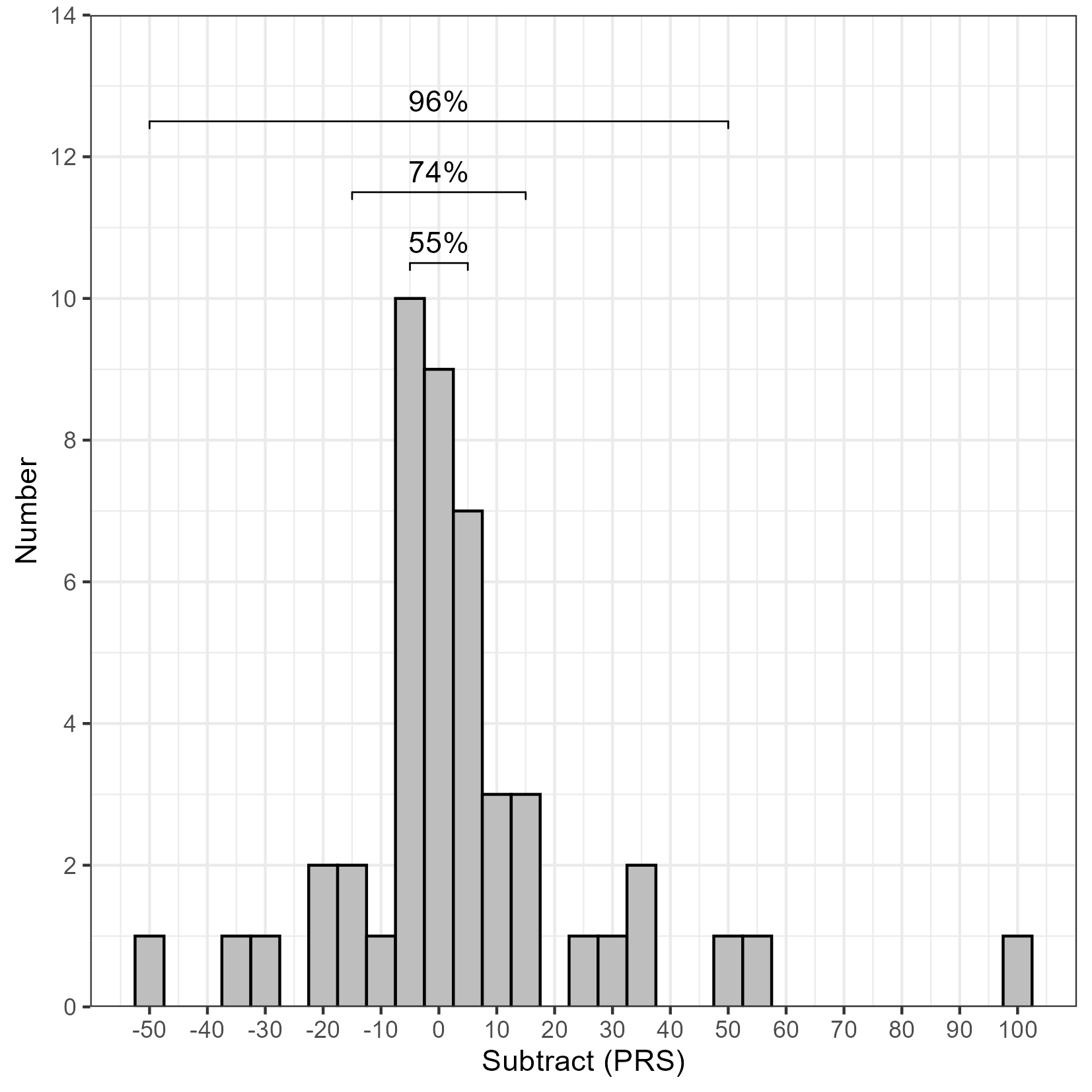


**Figure S11** Distribution of subtraction values in forty-seven sites, with brackets indicating classification boundaries: -5 to 5, -15 to 15, and -50 to 50. Numbers represent the percentage of sites within each coverage range.


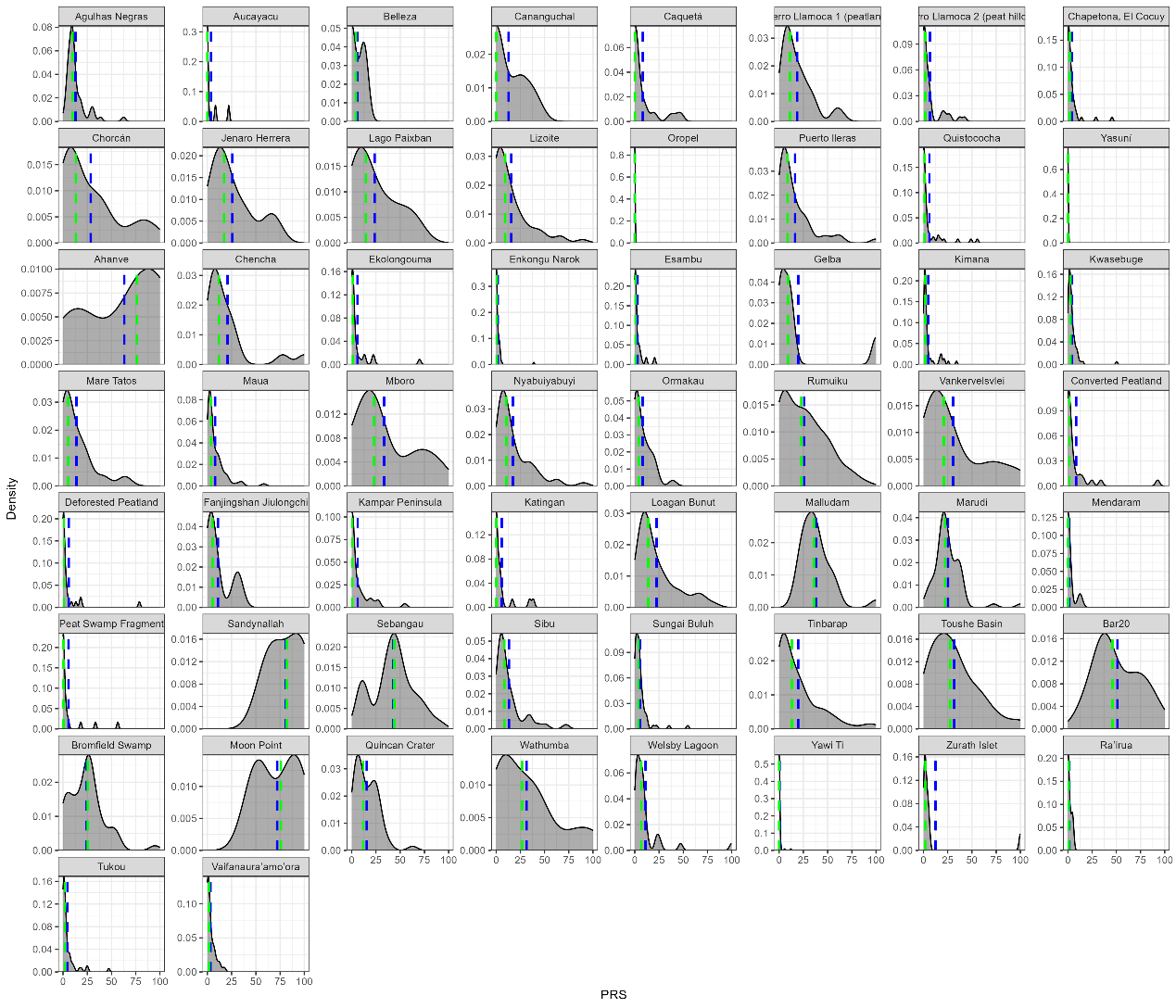


**Figure S12** Density plot for each peat site. The mean (blue) and median (green) values in each charcoal PRS value are shown.


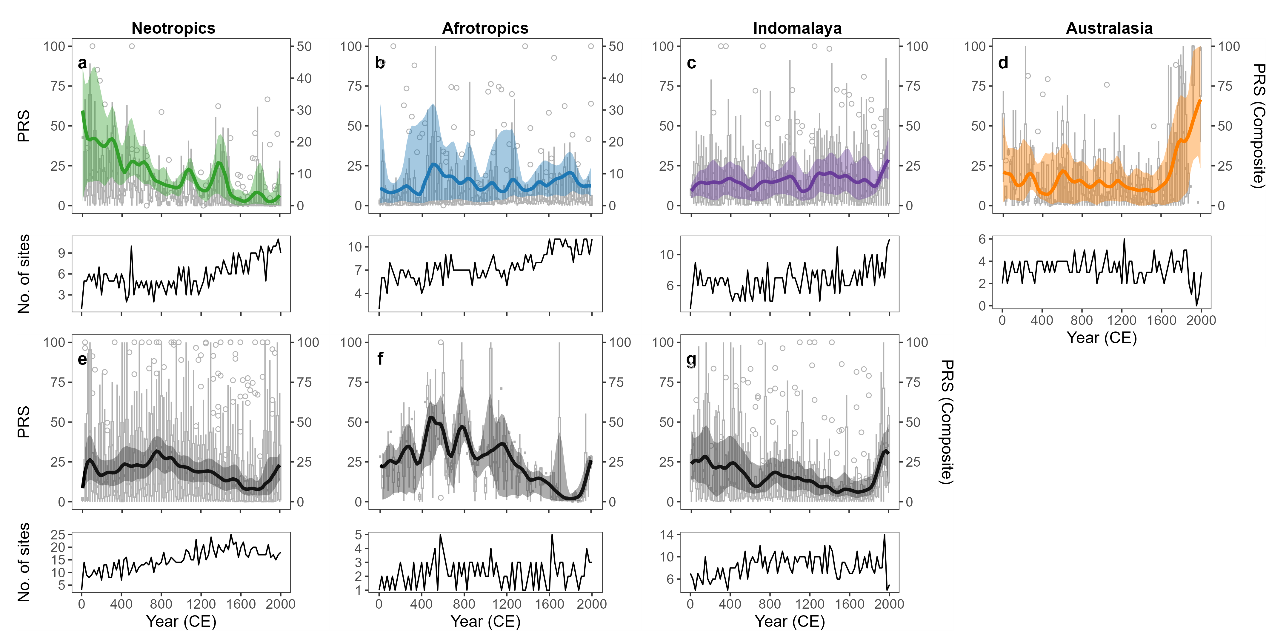


**Figure S13** Peatland burning (a-d) and landscape burning (e-g) in Neotropics (a, e), Afrotropics (b, f), Indomalaya (c, g) and Australasia (d, g) over the last 2,000 years. Note: the PRS (Composite) values have been halved from the original scale in panel (a-b) for better visualisation.


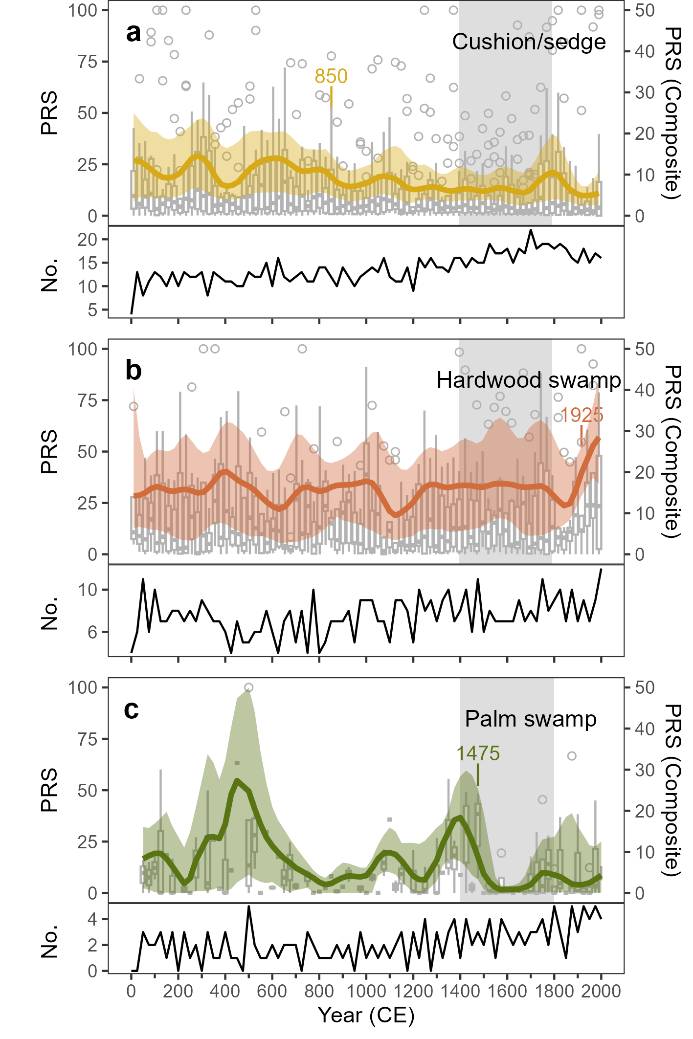


**Figure S14** **Peat burning history over the last 2,000 years for different ecosystem types.** (a) cushion/sedge peatlands, (b) hardwood swamps (c) and palm swamps. Composite curves were smoothed with a 200-yr window with 95% bootstrap confidence intervals (shaded area). Solid lines below each series indicate the number of sites (No.) contained in each non-overlapping 25-yr bin. The vertical shading represents the Little Ice Age.

***
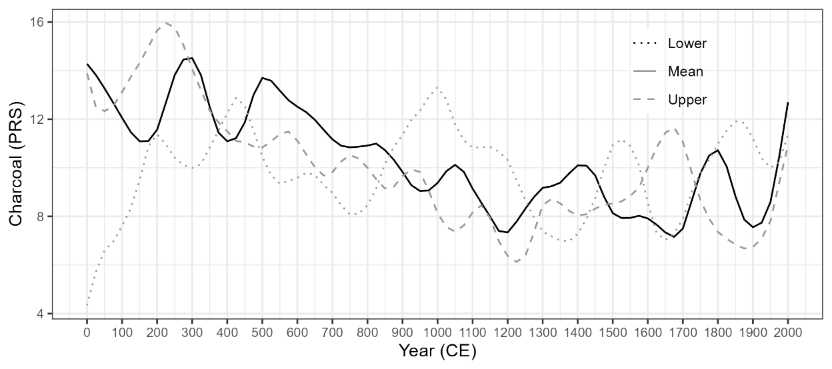
***

**Figure S15 Age uncertainty in age-depth models for tropical peatlands.** Peatland burning derived from mean age of age-depth models (solid, black) with uncertainty represented by the lower (dotted, grey) and upper (dashed, grey) age estimates.

**Table S1** **Detailed information on the compiled peatland sites in this study.** Regions are abbreviated as follows: NT-Neotropics; AT-Afrotropics; IM-Indomalaya; AA-Australasia and OC-Oceania. Database refers to relevant data sources and is abbreviated as follows: RPD-The Reading Palaeofire Database (Harrison et al., 2021); HOLO-HOLOPEATFIRE project in this study; PANGEA (Diepenbroek et al., 2002); SI-data from supplementary material of publication and Neotoma-Neotoma Paleoecology Database (Williams et al., 2018). Elevation data were obtained from SRTM elevation data for WorldClim 2.1(Fick et al., 2017).

| **Site Name** | **Region** | **Lat (°)** | **Lon (°)** | **Elevation (m)** | **Charcoal size** | **Country** | **Peatland type** | **Calibration curve** | **Contributor(s) of HOLO** | **Database** | **ref** |
| --- | --- | --- | --- | --- | --- | --- | --- | --- | --- | --- | --- |
| Agulhas Negras | NT | -22.39 | -44.67 | 2375 | >100µm | Brazil | cushion/sedge | SHCal20 |  | RPD | (Behling et al., 2020) |
| Aucayacu | NT | -3.93 | -74.39 | 123 | >1mm | Peru | other (pole forest) | 50:50 mixed | Graeme. T Swindles | HOLO | (Swindles et al., 2018) |
| Belleza | NT | 0.75 | -75.26 | 198 | >125µm | Colombia | palm swamp | 50:50 mixed | Yuwan Wang | HOLO | This study |
| Cananguchal | NT | -3.81 | -70.23 | 100 | >125µm | Colombia | palm swamp | 50:50 mixed | Yuwan Wang | HOLO | This study |
| Caquetá | NT | 1.13 | -75.43 | 210 | >125µm | Colombia | palm swamp | 50:50 mixed | Yuwan Wang | HOLO | This study |
| Cerro Llamoca 1 (peatland) | NT | -14.17 | -74.73 | 4285 | >125µm | Peru | cushion/sedge | SHCal20 | Karsten Schittek | HOLO | Unpublished charcoal data, (Schittek et al., 2015) |
| Cerro Llamoca 2 (peat hillock) | NT | -14.17 | -74.73 | 4285 | >125µm | Peru | cushion/sedge | SHCal20 | Karsten Schittek | HOLO | (Schittek et al., 2018) |
| Chapetona, El Cocuy | NT | 6.49 | -72.35 | 4125 | >125µm | Colombia | cushion/sedge | 50:50 mixed | Adam Benfield | HOLO | (Benfield et al., 2021) |
| Chorcán | NT | -23.05 | -65.23 | 4143 | >125µm | Argentina | cushion/sedge | 50:50 mixed | Karsten Schittek | HOLO | (Schittek, 2014) |
| Jenaro Herrera | NT | -4.96 | -73.67 | 133 | >125µm | Peru | palm swamp | 50:50 mixed | Yuwan Wang | HOLO | This study |
| Lago Paixban | NT | 17.8 | -90.12 | 209 | >125µm | Guatemala | cushion/sedge | IntCal20 | Lysanna Anderson, David Wahl | HOLO | (Anderson et al., 2016; Wahl et al., 2016; Anderson, 2025) |
| Lizoite | NT | -22.23 | -65.24 | 4465 | >125µm | Argentina | cushion/sedge | 50:50 mixed | Karsten Schittek | HOLO | (Schittek, 2014) |
| Oropel | NT | 9.38 | -82.37 | 12 | 200-1000µm | Panama | cushion/sedge | IntCal20 | Mariusz Gałka, Graeme. T Swindles | HOLO | Unpublished |
| Puerto lleras | NT | 3.26 | -73.42 | 241 | >125µm | Colombia | palm swamp | 50:50 mixed | Yuwan Wang | HOLO | This study |
| Quistococha | NT | -3.83 | -73.32 | 99 | >180µm | Peru | palm swamp | 50:50 mixed | Yuwan Wang | HOLO | This study |
| Yasuní | NT | -0.69 | -76.43 | 249 | >100µm | Ecuador | palm swamp | 50:50 mixed | Encarni Montoya | HOLO | Unpublished |
| Ahanve | AT | 6.43 | 2.77 | 4 | 100-200 | Nigeria | other (mangrove) | SHCal20 | Orijemie Emuobosa Akpo | HOLO | Unpublished |
| Chencha | AT | 6.3 | 37.57 | 2985 | >125µm | Ethiopia | cushion/sedge | 50:50 mixed | Femke Augustijns, Gert Verstraeten | HOLO | Unpublished |
| Ekolongouma | AT | 1.18 | 17.64 | 327 | >150µm | Republic of Congo | hardwood swamp | SHCal20 |  | PANGEA | (Garcin et al., 2022) |
| Enkongu Narok | AT | -2.7 | 37.26 | 1136 | >125µm | Kenya | cushion/sedge | 50:50 mixed | Esther Githumbi | HOLO | (Githumbi et al., 2018) |
| Esambu | AT | -2.71 | 37.55 | 1196 | >125µm | Kenya | cushion/sedge | 50:50 mixed | Esther Githumbi | HOLO | (Githumbi et al., 2018) |
| Gelba | AT | 6.04 | 37.46 | 2368 | >125µm | Ethiopia | cushion/sedge | 50:50 mixed | Femke Augustijns, Gert Verstraeten | HOLO | Unpublished |
| Kimana | AT | -2.75 | 37.52 | 1216 | >125µm | Kenya | cushion/sedge | 50:50 mixed | Esther Githumbi | HOLO | (Githumbi et al., 2018) |
| Kwasebuge | AT | -4.29 | 37.92 | 1949 | >125µm | Tanzania | cushion/sedge | 50:50 mixed | Colin Courtney-Mustaphi, Rob Marchant | HOLO | (Finch et al., 2017) |
| Mare Tatos | AT | -20.21 | 57.77 | 27 | >160µm | Mauritius | other (degraded) | SHCal20 |  | RPD | (Gosling et al., 2017) |
| Maua | AT | -3.13 | 37.43 | 3938 | >125µm | Tanzania | cushion/sedge | 50:50 mixed | Colin Courtney-Mustaphi, Rob Marchant | HOLO | (Mustaphi et al., 2021) |
| Mboro | AT | 15.15 | -16.89 | 13 | >160µm | Senegal | other (Niayes) | IntCal20 |  | SI | (Ndiaye et al., 2022) |
| Nyabuiyabuyi | AT | -0.44 | 35.8 | 2934 | >125µm | Kenya | cushion/sedge | 50:50 mixed | Esther Githumbi | HOLO | (Githumbi et al., 2021) |
| Ormakau | AT | -2.72 | 37.46 | 1174 | >125µm | Kenya | cushion/sedge | 50:50 mixed | Esther Githumbi | HOLO | (Githumbi et al., 2018) |
| Rumuiku | AT | -0.12 | 37.56 | 2178 | >125µm | Kenya | cushion/sedge | 50:50 mixed | Colin Courtney-Mustaphi, Rob Marchant, Esther Githumbi | HOLO | Unpublished charcoal data; (Rucina et al., 2009; Rucina, 2011) |
| Vankervelsvlei | AT | -34.01 | 22.9 | 167 | >125µm | South Africa | cushion/sedge | SHCal20 | Paul Strobel | HOLO | (Strobel et al., 2024) |
| Converted Peatland | IM | 3.87 | 113.71 | 11 | >150µm | Malaysia | hardwood swamp | 50:50 mixed | Lydia Cole | HOLO | (Cole et al., 2019) |
| Deforested Peatland | IM | 4.51 | 114.05 | 7 | >150µm | Malaysia | hardwood swamp | 50:50 mixed | Lydia Cole | HOLO | (Cole et al., 2019) |
| Fanjingshan Jiulongchi | IM | 27.9 | 108.69 | 1590 | >100µm | China | other (forest) | 50:50 mixed | Kunshan Bao, Yang Gao | HOLO | Unpublished |
| Kampar Peninsula | IM | 0.39 | 102.76 | 29 | >150µm | Indonesia | hardwood swamp | 50:50 mixed |  | PANGEA | (Hapsari et al., 2022) |
| Katingan | IM | -2.87 | 113.14 | 26 | >150µm | Indonesia | hardwood swamp | 50:50 mixed |  | PANGEA | (Hapsari et al., 2022) |
| Loagan Bunut | IM | 3.79 | 114.23 | 32 | >125µm | Malaysia | hardwood swamp | 50:50 mixed |  | PANGEA | (Yamamoto et al., 2021) |
| Malludam | IM | 1.48 | 111.17 | 20 | >125µm | Malaysia | hardwood swamp | 50:50 mixed |  | PANGEA | (Yamamoto et al., 2021) |
| Marudi | IM | 4.21 | 114.26 | 10 | >125µm | Malaysia | hardwood swamp | 50:50 mixed |  | PANGEA | (Yamamoto et al., 2021) |
| Mendaram | IM | 4.37 | 114.36 | 7 | >100µm | Brunei Darussalam | hardwood swamp | 50:50 mixed | René Dommain | HOLO | (Dommain et al., 2015) |
| Peat Swamp Fragment | IM | 4.36 | 114.01 | 13 | >150µm | Malaysia | hardwood swamp | 50:50 mixed | Lydia Cole | HOLO | (Cole et al., 2019) |
| Sandynallah | IM | 11.44 | 76.64 | 2186 | >125µm | India | cushion/sedge | 50:50 mixed | Ramya Bala Prabhakaran; Sarath Pullyottum Kavil; Raman Sukumar | HOLO | (Kavil et al., 2023) |
| Sebangau | IM | -2.32 | 113.9 | 20 | >125µm | Borneo | hardwood swamp | 50:50 mixed | Khairn Nisha Mohamed Ramdzan | HOLO | Unpublished |
| Sibu | IM | 2.18 | 111.86 | 35 | >250µm | Malaysia | hardwood swamp | 50:50 mixed |  | PANGEA | (Yamamoto et al., 2021) |
| Sungai Buluh | IM | -1.24 | 103.62 | 14 | >150µm | Indonesia | hardwood swamp | 50:50 mixed | K. Anggi Hapsari | HOLO | (Hapsari et al., 2017) |
| Tinbarap | IM | 4.05 | 114.25 | 31 | >125µm | Malaysia | hardwood swamp | 50:50 mixed |  | PANGEA | (Yamamoto et al., 2021) |
| Toushe Basin | IM | 23.83 | 120.9 | 654 | >125µm | China | hardwood swamp | 50:50 mixed |  | RPD | (Huang et al., 2020) |
| Bar20 | AA | -10.13 | 142.15 | 69 | >125µm | Australia | hardwood swamp | 50:50 mixed |  | RPD | (Rowe, 2006) |
| Bromfield Swamp | AA | -17.38 | 145.54 | 771 | >250µm | Australia | cushion/sedge | SHCal20 | Scott Mooney, Jess O'Donnell | HOLO | Unpublished charcoal data; (Roe, 2015) |
| Moon Point | AA | -25.22 | 153.06 | 7 | >250µm | Australia | cushion/sedge | SHCal20 | Patrick Moss | HOLO | (Moss et al., 2015) |
| Quincan Crater | AA | -17.3 | 145.58 | 753 | >250µm | Australia | cushion/sedge | SHCal20 | Scott Mooney, Jess O'Donnell | HOLO | Unpublished charcoal data; (Roe, 2015) |
| Wathumba | AA | -24.98 | 153.26 | 8 | >250µm | Australia | cushion/sedge | SHCal20 | Patrick Moss | HOLO | (Moss et al., 2015) |
| Welsby Lagoon | AA | -27.5 | 153.45 | 144 | >250µm | Australia | cushion/sedge | SHCal20 | John Tibby | HOLO | (Barr et al., 2017) |
| Yawi Ti | AA | -6.61 | 143.88 | 1099 | macro | Papua New Guinea | no information | 50:50 mixed |  | RPD | (Haberle, 2007) |
| Zurath Islet | AA | -10.28 | 142.1 | 9 | >125µm | Australia | cushion/sedge | 50:50 mixed |  | RPD | (Rowe, 2006) |
| Ra’irua | OC | -23.87 | -147.7 | 30 | >125µm | French Polynesia | hardwood swamp | SHCal20 |  | Neotoma | (Prebble et al., 2019) |
| Tukou | OC | -27.6 | -144.4 | 51 | >125µm | French Polynesia | cushion/sedge | SHCal20 |  | Neotoma | (Prebble et al., 2019) |
| Vaifanaura’amo’ora | OC | -17.63 | -149.6 | 582 | >125µm | French Polynesia | cushion/sedge | SHCal20 |  | Neotoma | (Prebble et al., 2016) |

**Table S2 Metadata for non-peat records contained in landscape burning from the Reading Palaeofire Database (Harrison et al., 2021).** Region refers to the relevant ecoregions (NT-Neotropics, AT-Afrotropics, IM-AA-Indomalaya and Australasia). Elevation data were obtained from SRTM elevation data for WorldClim 2.1 (Fick et al., 2017). -777777 in depositional context refers to the missing values in the database. Acronyms of data sources described in the Reading Palaeofire Database: GCD4 - Global Charcoal Database Version 4 (Power et al., 2008), Neotoma -Neotoma Paleoecology Database (Williams et al., 2018) and PANGEA (Diepenbroek et al., 2002).

| **ID** | **Region** | **Site name** | **Lat (°)** | **Lon (°)** | **Elevation (m)** | **Site type** | **Depositional context** | **Unit** | **Data source** |
| --- | --- | --- | --- | --- | --- | --- | --- | --- | --- |
| 315 | NT | La Yeguada | 8.45 | -80.85 | 650 | lacustrine | lake sediment | other | GCD4 |
| 418 | NT | Salitre | -19.00 | -46.76 | 970 | terrestrial, soil | soil | % dry weight | GCD4 |
| 485 | NT | Lago Verde | 18.61 | -95.34 | 149 | lacustrine, volcanic lake | lake sediment | fragments 125-250 μm cm^-2^ yr^-1^ | GCD4 |
| 779 | NT | Laguna Verde | 13.89 | -89.78 | 1600 | lacustrine | lake sediment | % of pollen sum >25 μm | GCD4 |
| 780 | NT | Laguna Cuzcachapa | 13.98 | -89.68 | 709 | lacustrine | lake sediment | % of pollen sum >25 μm | GCD4 |
| 782 | NT | Laguna Santa Elena | 8.56 | -82.56 | 1100 | lacustrine | lake sediment | % of pollen sum >25 μm | GCD4 |
| 783 | NT | Marcacocha | -13.21 | -72.20 | 3355 | lacustrine | lake sediment | % of pollen sum >25 μm | GCD4 |
| 785 | NT | Laguna Pompal | 18.37 | -94.95 | 700 | lacustrine | lake sediment | mm^-2^ cm^-2^ yr^-1^ | GCD4 |
| 827 | NT | Laguna Bonillita | 9.99 | -83.61 | 450 | lacustrine | lake sediment | % of pollen sum >25 μm | GCD4 |
| 835 | NT | Pie de Gigante | 11.38 | -86.03 | 33 | coastal | -777777 | fragments <150 μm (concentration) | GCD4 |
| 847 | NT | Yaguaru | -15.60 | -63.21 | 195 | lacustrine | lake sediment | fragments <50 μm (concentration) | GCD4 |
| 2029 | NT | Laguna EkNaab | 17.42 | -89.24 | 160 | lacustrine | lake sediment | fragments >250 μm ml^-1^ | Neotoma |
| 2031 | NT | Laguna Cocha Caranga | -4.05 | -79.16 | 2710 | lacustrine | lake sediment | fragments 10-125 μm (count) | Neotoma |
| 2032 | NT | Laguna Cocha Caranga | -4.05 | -79.16 | 2700 | lacustrine | soil | fragments 10-125 μm (count) | Neotoma |
| 2034 | NT | Lake Refugio1 | -13.09 | -71.71 | 3401 | lacustrine | lake sediment | area >180 μm mm^-2^ ml^-1^ | Neotoma |
| 2035 | NT | Lake Refugio2 | -13.09 | -71.71 | 3406 | lacustrine | lake sediment | area >180 μm mm^-2^ ml^-1^ | Neotoma |
| 2036 | NT | Lake Refugio3 | -13.10 | -71.70 | 3404 | lacustrine | lake sediment | area >180 μm mm^-2^ ml^-1^ | Neotoma |
| 2038 | NT | Lake Acarabixi | -0.35 | -64.50 | 33 | lacustrine, fluvial origin | fluvial/alluvial sediment | fragments >10 μm cm^-2^ yr^-1^ | Neotoma |
| 2039 | NT | Lake Acarabixi | -0.35 | -64.50 | 33 | lacustrine, fluvial origin | fluvial/alluvial sediment | fragments >100 μm cm^-2^ yr^-1^ | Neotoma |
| 2046 | NT | Laguna Rabadilla de Vaca | -4.26 | -79.11 | 3312 | lacustrine | lake sediment | fragments 10-150 μm cm^-2^ yr^-1^ | Neotoma |
| 2047 | NT | Laguna Zurita | -3.97 | -79.12 | 2590 | lacustrine | lake sediment | fragments 10-150 μm (count) | Neotoma |
| 2048 | NT | Estacion Cientifica San Francisco Refugio | -3.98 | -79.07 | 2520 | terrestrial, soil | soil | fragments 10-150 μm (count) | Neotoma |
| 2050 | NT | Estacion Cientifica San Francisco Cerro de Consuelo | -4.00 | -79.06 | 3155 | terrestrial, soil | soil | fragments 10-150 μm (count) | Neotoma |
| 2051 | NT | Cenote Kail | 16.00 | -91.55 | 1534 | lacustrine | lake sediment | fragments 2-150 μm cm^-2^ yr^-1^ | Neotoma |
| 2052 | NT | Cenote Kail | 16.00 | -91.55 | 1534 | lacustrine | lake sediment | fragments >150 μm cm^-2^ yr^-1^ | Neotoma |
| 2161 | NT | Lake Pacucha | -13.61 | -73.50 | 3095 | lacustrine | lake sediment | mm^-2^ cm^-3^ | PANGAEA |
| 2301 | NT | Botucatu | -23.18 | -48.00 | 500 | terrestrial, soil | soil | % dry weight | GCD4 |
| 2305 | NT | Laguna Charco Verde | 11.47 | -85.63 | 33 | lacustrine, volcanic lake | lake sediment | fragments 5-150 μm (concentration) | GCD4 |
| 2310 | NT | El Patia | 2.03 | -77.00 | 760 | lacustrine | lake sediment | % dry weight | GCD4 |
| 2315 | NT | Gentry Lake | -12.17 | -69.09 | 270 | lacustrine | lake sediment | point count >20 μm cm^-2^ cm^-3^ | GCD4 |
| 2321 | NT | La-Teta | 3.08 | -76.53 | 1020 | lacustrine | lake sediment | % dry weight | GCD4 |
| 2322 | NT | Lago Chirripo | 9.48 | -83.50 | 3520 | lacustrine | lake sediment | pollen concentration μm^-2^ (area:pollen ratio) | GCD4 |
| 2324 | NT | Lago de las Morrenas | 9.48 | -83.48 | 3480 | lacustrine | lake sediment | pollen concentration μm^-2^ (area:pollen ratio) | GCD4 |
| 2325 | NT | Lago do Pires | -17.95 | -42.21 | 390 | lacustrine | lake sediment | % of pollen sum 5-50 μm | GCD4 |
| 2327 | NT | Lagoa Nova | -17.95 | -42.20 | 390 | lacustrine | lake sediment | fragments 5-50 μm (concentration) | GCD4 |
| 2328 | NT | Laguna Chorreras | -2.75 | -79.16 | 3700 | lacustrine, glacial origin | lake sediment | fragments 30-100 μm (concentration) | GCD4 |
| 2329 | NT | Laguna Chorreras | -2.75 | -79.16 | 3700 | lacustrine, glacial origin | lake sediment | fragments >100 μm cm^-3^ | GCD4 |
| 2330 | NT | Laguna de Chochos | -7.63 | -77.47 | 3285 | lacustrine, glacial origin | lake sediment | fragments <50 μm (concentration) | GCD4 |
| 2331 | NT | Laguna Las Margaritas | 3.38 | -73.43 | 290 | lacustrine, fluvial origin | lake sediment | fragments >10 μm (concentration) | GCD4 |
| 2332 | NT | Laguna Llana del Espino | 13.95 | -89.52 | 700 | lacustrine, tectonic origin | lake sediment | % of pollen sum >25 μm | GCD4 |
| 2333 | NT | Laguna Metapan | 14.30 | -89.48 | 450 | lacustrine, volcanic lake | lake sediment | fragments >150 μm cm^-3^ | GCD4 |
| 2335 | NT | Laguna Volcan | 8.75 | -82.68 | 1500 | lacustrine, volcanic lake | lake sediment | fragments cm^-3^ | GCD4 |
| 2337 | NT | Laguna Zoncho | 8.81 | -82.96 | 1190 | lacustrine, tectonic origin | lake sediment | fragments >5 μm g^-1^ | GCD4 |
| 2338 | NT | Lake Chalalan | -14.42 | -67.92 | 330 | lacustrine | lake sediment | mm^-2^ cm^-3^ | GCD4 |
| 2345 | NT | Lake Nicaragua | 11.76 | -85.87 | 30 | lacustrine, tectonic origin | lake sediment | fragments >150 μm cm^-3^ | GCD4 |
| 2347 | NT | Lake Santa Rosa | -14.47 | -67.87 | 350 | lacustrine | lake sediment | mm^-2^ cm^-3^ | Neotoma |
| 2350 | NT | Lake Titicaca | -16.21 | -69.21 | 3810 | lacustrine, tectonic origin | lake sediment | mm^-2^ cm^-3^ | GCD4 |
| 2357 | NT | Parker Lake | -12.14 | -69.02 | 276 | lacustrine | lake sediment | mm^-2^ cm^-3^ | GCD4 |
| 2362 | NT | Quilichao | 3.10 | -76.51 | 1020 | lacustrine | lake sediment | % dry weight | GCD4 |
| 2368 | NT | Surucucho | -3.06 | -78.00 | 3180 | lacustrine | lake sediment | fragments <25 μm (concentration) | GCD4 |
| 2369 | NT | Surucucho | -3.06 | -78.00 | 3180 | lacustrine | lake sediment | fragments >25 μm (concentration) | GCD4 |
| 2372 | NT | Vargas Lake | -12.37 | -68.89 | 246 | lacustrine | lake sediment | mm^-2^ cm^-3^ | GCD4 |
| 2374 | NT | Werth Lake | -11.74 | -69.23 | 302 | lacustrine | lake sediment | mm^-2^ cm^-3^ | GCD4 |
| 2408 | NT | Lake Huila | -0.42 | -78.02 | 2608 | lacustrine, volcanic lake | lake sediment | fragments <100 μm (count) | Neotoma |
| 2409 | NT | Lake Huila | -0.42 | -78.02 | 2608 | lacustrine, volcanic lake | lake sediment | fragments >100 μm (count) | Neotoma |
| 2412 | NT | Lake Kumpak^a^ | -2.84 | -77.96 | 333 | lacustrine, volcanic lake | lake sediment | Other (mm^3^ cm^-3^) | Neotoma |
| 2415 | NT | Lake Aljojuca | 19.09 | -97.53 | 2376 | lacustrine, volcanic lake | lake sediment | fragments >100 μm (concentration) | author |
| 209 | AT | Masoko | -9.33 | 33.75 | 770 | lacustrine, volcanic lake | lake sediment | fragments >125 μm cm^-3^ | GCD4 |
| 210 | AT | Masoko | -9.33 | 33.75 | 770 | lacustrine, volcanic lake | lake sediment | fragments >150 μm g^-1^ | GCD4 |
| 747 | AT | Hardibo | 11.23 | 39.76 | 2150 | lacustrine | lake sediment | mm^-2^ cm^-3^ | GCD4 |
| 748 | AT | Hayk | 11.35 | 39.71 | 1920 | lacustrine | lake sediment | mm^-2^ cm^-3^ | GCD4 |
| 963 | AT | Lake Doukoulou | 4.25 | 18.42 |  | lacustrine | -777777 | fragments >160 μm cm^-2^ yr^-1^ | GCD4 |
| 964 | AT | Lake Gbali | 4.82 | 18.26 | 386 | lacustrine | -777777 | fragments >160 μm cm^-2^ yr^-1^ | GCD4 |
| 966 | AT | Lake Nguengue | 3.77 | 18.12 | 385 | lacustrine | -777777 | fragments >160 μm cm^-2^ yr^-1^ | GCD4 |
| 1000 | AT | Garba Guracha | 6.88 | 39.87 | 3950 | lacustrine | -777777 | fragments cm^-2^ yr^-1^ | GCD4 |
| 2219 | AT | Mobutu Sese Seko Lake Albert | 1.83 | 31.16 | 619 | lacustrine, tectonic origin | lake sediment | fragments cm^-3^ | GCD4 |
| 2220 | AT | Lake Rutundu | -0.04 | 37.46 | 3140 | lacustrine | lake sediment | <180 μm x 10^4 relective graminoid cuticle area | GCD4 |
| 2229 | AT | Sacred Lake | 0.04 | 37.52 | 2350 | lacustrine | lake sediment | area 45-95 μm μm^-2^ cm^-3^ | GCD4 |
| 125 | IM-AA | Huguangyan Maar Lake | 21.15 | 110.28 | 87.6 | lacustrine | lake sediment | area (concentration) | GCD4 |
| 509 | IM-AA | Talita Kupai | -10.10 | 142.12 | 33 | coastal, estuarine | soil | fragments (concentration) | GCD4 |
| 510 | IM-AA | Waruid | -10.40 | 142.09 | 5 | coastal | soil | fragments >125 μm cm^-3^ | GCD4 |
| 588 | IM-AA | Paoay Lake | 18.20 | 120.54 | 15 | lacustrine | lake sediment | fragments ml^-1^ | GCD4 |
| 594 | IM-AA | Ajkwa 1 | -4.86 | 136.96 | 1.3 | coastal, estuarine | estuarine sediment | % of pollen sum | GCD4 |
| 595 | IM-AA | Ajkwa 2 | -4.86 | 136.96 | 1.3 | coastal, estuarine | estuarine sediment | % of pollen sum | GCD4 |
| 596 | IM-AA | Ajkwa 3 | -4.86 | 136.96 | 1.3 | coastal, estuarine | estuarine sediment | % of pollen sum | GCD4 |
| 597 | IM-AA | Ajkwa 4 | -4.86 | 136.96 | 1.3 | coastal, estuarine | estuarine sediment | % of pollen sum | GCD4 |
| 598 | IM-AA | Ajkwa 5 | -4.86 | 136.96 | 1.3 | coastal, estuarine | estuarine sediment | % of pollen sum | GCD4 |
| 603 | IM-AA | Hogayaku | -3.98 | 137.38 | 3580 | lacustrine, glacial origin | lake sediment | cm^-2^ cm^-3^ | GCD4 |
| 661 | IM-AA | Aguai Ramata | -6.56 | 145.21 | 1950 | lacustrine, volcanic lake | lake sediment | fragments cm^-3^ | GCD4 |
| 662 | IM-AA | Sondambile | -6.34 | 147.11 | 2850 | lacustrine, glacial origin | lake sediment | fragments cm^-3^ | GCD4 |
| 666 | IM-AA | Wanum | -6.63 | 146.79 | 35 | lacustrine | lake sediment | fragments cm^-3^ | GCD4 |
| 797 | IM-AA | Lynchs Crater | -17.36 | 145.70 | 760 | lacustrine, drained lake | lake sediment | fragments cm^-3^ | GCD4 |
| 1954 | IM-AA | Xingyun Lake | 24.33 | 102.77 | 1723 | lacustrine | lake sediment | fragments cm^-2^ yr^-1^ | digitized from publication |
| 1996 | IM-AA | Taibai Lake | 30.00 | 115.81 | 10 | lacustrine | lake sediment | fragments >125 μm cm^-3^ | author |
| 1997 | IM-AA | Taibai Lake | 30.00 | 115.81 | 10 | lacustrine | lake sediment | fragments <125 μm cm^-2^ cm^-3^ | author |
| 2143 | IM-AA | ODP Site 820 | -16.63 | 146.30 | -280 | marine | marine sediment | fragments cm^-3^ | GCD3 |
| 2162 | IM-AA | Native Companion Lagoon | -27.67 | 153.41 | 20 | lacustrine | lake sediment | fragments >10 μm cm-^3^ | PANGAEA |
| 2230 | IM-AA | SONNE95 | 20.11 | 117.38 | -1727 | marine | marine sediment | fragments cm^-3^ | GCD4 |
| 2298 | IM-AA | Allom Lake | -25.23 | 153.16 | 100 | lacustrine | lake sediment | fragments 120-250 μm cm^-2^ yr^-1^ | GCD4 |
| 2339 | IM-AA | Old Lake Coomboo Depression | -25.23 | 153.18 | 86.4 | lacustrine | lake sediment | mm^-2^ cm^-3^ | GCD4 |
| 2340 | IM-AA | Lake Euramoo | -17.15 | 145.62 | 718 | lacustrine, volcanic lake | lake sediment | fragments >125 μm cm^-2^ yr^-1^ | GCD4 |

**Table S3 Linear regression on peatland burning with age for different periods and subregions based on the composite curve.** Significant codes for predictor: *p* < 0.001 (***); *p* <0.01 (**); *p* <0.05 (*).

|  | **Significance** | **Predictor coefficient**  **(Intercept)** | **Predictor coefficient**  **(age)** | **Adjusted R^2^** |
| --- | --- | --- | --- | --- |
| **Tropical region** |  |  |  |  |
| 0-850 CE | *p* < 0.01 | 13.2*** | -0.002** | 0.18 |
| 850-1900 CE | *p* = 0.10 | 10.1*** | -0.001 | 0.04 |
| 1900-2000 CE | *p* < 0.05 | -121.6* | 0.067* | 0.95 |
| **Neotropical realm** |  |  |  |  |
| 0-850 CE | *p* < 0.001 | 24.1*** | -0.021*** | 0.86 |
| 850-1900 CE | *p* < 0.001 | 13.8*** | -0.006*** | 0.25 |
| 1900-2000 CE | *p* < 0.05 | -45.9* | 0.025* | 0.96 |
| **Afrotropical realm** |  |  |  |  |
| 0-850 CE | *p* < 0.001 | 4.2*** | 0.007*** | 0.38 |
| 850-1900 CE | *p* < 0.001 | 2.7** | 0.003*** | 0.31 |
| 1900-2000 CE | *p* = 0.10 | 2.3 | 0.002 | 0.73 |
| **Indomalayan realm** |  |  |  |  |
| 0-850 CE | *p* = 0.78 | 13.8*** | 0.000 | -0.03 |
| 850-1900 CE | *p* = 0.86 | 15.7*** | 0.000 | -0.02 |
| 1900-2000 CE | *p* < 0.01 | -221.4** | 0.125** | 0.99 |
| **Australasian realm** |  |  |  |  |
| 0-850 CE | *p* = 0.42 | 16.3*** | -0.003 | -0.01 |
| 850-1900 CE | *p* < 0.001 | -11.3 | 0.021*** | 0.36 |
| 1900-2000 CE | *p* < 0.01 | -277.6* | 0.172** | 0.97 |

**Table S4 Multiple linear regression and linear mixed effects regression for different periods.** Bold font indicates statistically significant predictor variables.

| **Period** | **Predictors** | **Equations** | R^2^ | p | **dAICc** | **Weight** |
| --- | --- | --- | --- | --- | --- | --- |
| 0-2000 CE (n=58) | **lon + bio_14** | 2.171+0.005*lon-0.007*bio_14 | 0.24 | <0.001 | 0 | 0.726 |
|  | **lon** + bio_4+**bio_14** | 2.045+0.004*lon+0.001*bio_4-0.006*bio_14 | 0.23 | <0.001 | 2.1 | 0.251 |
|  | bio_14 + (1 \| Region) | |  |  | 6.9 | 0.023 |
| 0-850 CE (n=48) | **bio_15** | 1.078+0.014*bio_15 | 0.16 | <0.01 | 0 | 0.46 |
|  | bio_4 + **bio_15** | 0.949+0.002*bio_4+0.011*bio_15 | 0.18 | <0.01 | 0.3 | 0.4 |
|  | bio_15 + (1 \| Region) | |  |  | 2.4 | 0.14 |
| 850-1900 CE (n=55) | **bio_4** | 1.353+0.004*bio_4 | 0.07 | <0.05 |  |  |
| 1900-2000 CE (n=48) | **lon** | 1.546+0.010*lon | 0.32 | <0.001 | 0 | 0.51 |
|  | **lon** + MCWD | 1.687+0.010*lon+0.00005*MCWD | 0.34 | <0.001 | 0.2 | 0.471 |
|  | MCWD + (1 \| Region) | |  |  | 6.5 | 0.019 |

lon: longitude; bio_4: temperature seasonality (standard deviation ×100); bio_14: precipitation of the driest month; bio_15: = precipitation seasonality (coefficient of variation); MCWD: maximum cumulative water deficit; Region: Neotropical, Afrotropical, Indomalayan, Australasian and Oceanian ecoregions.

USGS Disclaimer: Any use of trade, firm, or product names is for descriptive purposes only and does not imply endorsement by the U.S. Government.

**References:**

Anderson, L. (2025). Sub-Fossil Charcoal Particle Counts and Radiocarbon Age Determinations from a 2003 Core Recovered in a Perennial Wetland at Paixban, Northern Guatemala: U.S. Geological Survey data release. https://doi.org/10.5066/p15ibcso.

Anderson, L., & Wahl, D. (2016). Two Holocene paleofire records from Peten, Guatemala: Implications for natural fire regime and prehispanic Maya land use. Global and Planetary Change, 138, 82-92. https://doi.org/10.1016/j.gloplacha.2015.09.012.

Barr, C., Tibby, J., Moss, P. T., Halverson, G. P., Marshall, J. C., McGregor, G. B., & Stirling, E. (2017). A 25,000-year record of environmental change from Welsby Lagoon, North Stradbroke Island, in the Australian subtropics. Quaternary International, 449, 106-118. https://doi.org/10.1016/j.quaint.2017.04.011.

Behling, H., Jantz, N., & Safford, H. D. (2020). Mid-and late Holocene vegetation, climate and fire dynamics in the Serra do Itatiaia, Rio de Janeiro State, southeastern Brazil. Review of Palaeobotany and Palynology, 274, 104152. https://doi.org/10.1016/j.revpalbo.2019.104152.

Benfield, A. J., Yu, Z., & Benavides, J. C. (2021). Environmental controls over Holocene carbon accumulation in Distichia muscoides-dominated peatlands in the eastern Andes of Colombia. Quaternary Science Reviews, 251, 106687. https://doi.org/10.1016/j.quascirev.2020.106687.

Cole, L. E., Bhagwat, S. A., & Willis, K. J. (2019). Fire in the swamp forest: palaeoecological insights into natural and human-induced burning in intact tropical peatlands. Frontiers in Forests and Global Change, 2, 48. https://doi.org/10.3389/ffgc.2019.00048.

Diepenbroek, M., Grobe, H., Reinke, M., Schindler, U., Schlitzer, R., Sieger, R., & Wefer, G. (2002). PANGAEA—an information system for environmental sciences. Computers & Geosciences, 28(10), 1201-1210. https://doi.org/10.1016/S0098-3004(02)00039-0.

Dommain, R., Cobb, A. R., Joosten, H., Glaser, P. H., Chua, A. F., Gandois, L., . . . Harvey, C. F. (2015). Forest dynamics and tip‐up pools drive pulses of high carbon accumulation rates in a tropical peat dome in Borneo (Southeast Asia). Journal of Geophysical Research: Biogeosciences, 120(4), 617-640. https://doi.org/10.1002/2014JG002796.

Fick, S. E., & Hijmans, R. J. (2017). WorldClim 2: new 1‐km spatial resolution climate surfaces for global land areas. International Journal of Climatology, 37(12), 4302-4315. https://doi.org/10.1002/joc.5086.

Finch, J., Marchant, R., & Courtney Mustaphi, C. J. (2017). Ecosystem change in the South Pare Mountain bloc, Eastern Arc Mountains of Tanzania. The Holocene, 27(6), 796-810.

Garcin, Y., Schefuß, E., Dargie, G. C., Hawthorne, D., Lawson, I. T., Sebag, D., . . . Ifo, S. A. (2022). Hydroclimatic vulnerability of peat carbon in the central Congo Basin. Nature, 1-6. https://doi.org/10.1038/s41586-022-05389-3.

Githumbi, E. N., Courtney Mustaphi, C. J., & Marchant, R. (2021). Late Pleistocene and Holocene Afromontane vegetation and headwater wetland dynamics within the Eastern Mau Forest, Kenya. Journal of Quaternary Science, 36(2), 239-254. https://doi.org/10.1002/jqs.3267.

Githumbi, E. N., Kariuki, R., Shoemaker, A., Courtney-Mustaphi, C. J., Chuhilla, M., Richer, S., . . . Marchant, R. (2018). Pollen, people and place: multidisciplinary perspectives on ecosystem change at Amboseli, Kenya. Frontiers in Earth Science, 5, 113. https://doi.org/10.3389/feart.2017.00113.

Gosling, W. D., de Kruif, J., Norder, S. J., de Boer, E. J., Hooghiemstra, H., Rijsdijk, K. F., & McMichael, C. N. (2017). Mauritius on fire: Tracking historical human impacts on biodiversity loss. Biotropica, 49(6), 778-783. https://doi.org/10.1111/btp.12490.

Haberle, S. (2007). Was the early Holocene characterised by" El Niño-like" or" La Niña-like" conditions? Evidence from terrestrial archives in the western Pacific Warm Pool region. Paper presented at the Quaternary International 167e168 (Supplement (XVII INQUA Congress)).

Hapsari, K. A., Biagioni, S., Jennerjahn, T. C., Reimer, P. M., Saad, A., Achnopha, Y., . . . Behling, H. (2017). Environmental dynamics and carbon accumulation rate of a tropical peatland in Central Sumatra, Indonesia. Quaternary Science Reviews, 169, 173-187. https://doi.org/10.1016/j.quascirev.2017.05.026.

Hapsari, K. A., Jennerjahn, T., Nugroho, S. H., Yulianto, E., & Behling, H. (2022). Sea level rise and climate change acting as interactive stressors on development and dynamics of tropical peatlands in coastal Sumatra and South Borneo since the Last Glacial Maximum. Global Change Biology, 28(10), 3459-3479. https://doi.org/10.1111/gcb.16131.

Harrison, S. P., Villegas-Diaz, R., Cruz-Silva, E., Gallagher, D., Kesner, D., Lincoln, P., . . . Ali, A. (2021). The Reading Palaeofire database: an expanded global resource to document changes in fire regimes from sedimentary charcoal records. Earth System Science Data Discussions, 1-30. https://doi.org/10.5194/essd-14-1109-2022.

Huang, Z., Ma, C., Chyi, S. J., Tang, L., & Zhao, L. (2020). Paleofire, vegetation, and climate reconstructions of the middle to late Holocene from lacustrine sediments of the Toushe Basin, Taiwan. Geophysical Research Letters, 47(20), e2020GL090401. https://doi.org/10.1029/2020GL090401.

Kavil, S. P., Bala, P. R., Ghosh, D., Kumar, P., & Sukumar, R. (2023). Climate change and the migration of a pastoralist people c. 3500 cal. years BP inferred from palaeofire and lipid biomarker records in the montane Western Ghats, India. Environmental Archaeology, 28(3), 192-206. https://doi.org/10.1080/14614103.2021.1959188.

Moss, P., Tibby, J., Shapland, F., Fairfax, R., Stewart, P., Barr, C., . . . Sloss, C. (2015). Patterned fen formation and development from the Great Sandy Region, south-east Queensland, Australia. Marine and Freshwater Research, 67(6), 816-827.

Mustaphi, C. J. C., Kinyanjui, R., Shoemaker, A., Mumbi, C., Muiruri, V., Marchant, L., . . . Marchant, R. (2021). A 3000-year record of vegetation changes and fire at a high-elevation wetland on Kilimanjaro, Tanzania. Quaternary Research, 99, 34-62. https://doi.org/10.1017/qua.2020.76.

Ndiaye, A., Bentaleb, I., Favier, C., Fourel, F., Sebag, D., Fall, M., . . . Diouf, B. (2022). Reconstruction of the holocene climate and environmental changes of Niayes peat bog in northern coast of Senegal (NW Africa) based on stable isotopes and charcoals analysis. Quaternary Science Reviews, 289, 107609. https://doi.org/10.1016/j.quascirev.2022.107609.

Power, M. J., Marlon, J., Ortiz, N., Bartlein, P. J., Harrison, S. P., Mayle, F. E., . . . Cordova, C. (2008). Changes in fire regimes since the Last Glacial Maximum: an assessment based on a global synthesis and analysis of charcoal data. Climate Dynamics, 30(7-8), 887-907. https://doi.org/10.1007/s00382-007-0334-x.

Prebble, M., Anderson, A. J., Augustinus, P., Emmitt, J., Fallon, S. J., Furey, L. L., . . . Matthews, P. J. (2019). Early tropical crop production in marginal subtropical and temperate Polynesia. Proceedings of the National Academy of Sciences, 116(18), 8824-8833. https://doi.org/10.1073/pnas.1821732116

Prebble, M., Whitau, R., Meyer, J. Y., Sibley‐Punnett, L., Fallon, S., & Porch, N. (2016). Abrupt late Pleistocene ecological and climate change on Tahiti (French Polynesia). Journal of Biogeography, 43(12), 2438-2453. https://doi.org/10.1111/jbi.12807.

Roe, J. (2015). High-resolution climate reconstruction for the last 3000 years from lake sediments in tropical Queensland. (PhD Thesis). Univeristy of New South Wale, Sydney, Australia.

Rowe, C. (2006). A Holocene history of vegetation change in the western Torres Strait region, Queensland, Australia. Quaternary Australasia, 24, 14-15.

Rucina, S. (2011). Kenyan ecosystem dynamics: perspectives from high and low altitude ecosystems. (PhD thesis). Universiteit van Amsterdam, Amsterdam, Netherlands.

Rucina, S. M., Muiruri, V. M., Kinyanjui, R. N., McGuiness, K., & Marchant, R. (2009). Late Quaternary vegetation and fire dynamics on Mount Kenya. Palaeogeography, Palaeoclimatology, Palaeoecology, 283(1-2), 1-14. https://doi.org/10.1016/j.palaeo.2009.08.008.

Schittek, K. (2014). Cushion peatlands in the high Andes of northwestern Argentina as archives for palaeoenvironmental research. Dissertationes Botanicae.

Schittek, K., Forbriger, M., Berg, D., Hense, J., Schäbitz, F., & Eitel, B. (2018). Last millennial environmental dynamics in the western Peruvian Andes inferred from the development of a cushion-plant peat hillock. Perspectives in Plant Ecology, Evolution and Systematics, 30, 115-124. https://doi.org/10.1016/j.ppees.2017.09.002.

Schittek, K., Forbriger, M., Mächtle, B., Schäbitz, F., Wennrich, V., Reindel, M., & Eitel, B. (2015). Holocene environmental changes in the highlands of the southern Peruvian Andes (14 S) and their impact on pre-Columbian cultures. Climate of the Past, 11(1), 27-44. https://doi.org/10.5194/cp-11-27-2015.

Strobel, P., Henning, T., Bliedtner, M., Mosher, S. G., Rahimova, H., Haberzettl, T., . . . Zech, M. (2024). Holocene fire dynamics and their climatic controls on the southern Cape coast of South Africa-A 7.2 ka multi-proxy record from the peatland Vankervelsvlei. Quaternary Science Reviews, 325, 108464. https://doi.org/10.1016/j.quascirev.2023.108464.

Swindles, G. T., Morris, P. J., Whitney, B., Galloway, J. M., Gałka, M., Gallego‐Sala, A., . . . Amesbury, M. J. (2018). Ecosystem state shifts during long‐term development of an Amazonian peatland. Global Change Biology, 24(2), 738-757. https://doi.org/10.1111/gcb.13950

Wahl, D., Hansen, R. D., Byrne, R., Anderson, L., & Schreiner, T. (2016). Holocene climate variability and anthropogenic impacts from Lago Paixban, a perennial wetland in Peten, Guatemala. Global and Planetary Change, 138, 70-81. https://doi.org/10.1016/j.gloplacha.2015.09.011.

Williams, J. W., Grimm, E. C., Blois, J. L., Charles, D. F., Davis, E. B., Goring, S. J., . . . Arroyo-Cabrales, J. (2018). The Neotoma Paleoecology Database, a multiproxy, international, community-curated data resource. Quaternary Research, 89(1), 156-177. https://doi.org/10.1017/qua.2017.105.

Yamamoto, M., Kikuchi, T., Sakurai, H., Hayashi, R., Seki, O., Omori, T., . . . Melling, L. (2021). Tropical Western Pacific hydrology during the last 6,000 years based on wildfire charcoal records from Borneo. Geophysical Research Letters, 48(18). https://doi.org/10.1029/2021gl093832.
